# Infrastructure and operating processes of PIONEER, the HDR-UK Data Hub in Acute Care and the workings of the Data Trust Committee: a protocol paper

**DOI:** 10.1136/bmjhci-2020-100294

**Published:** 2021-04-13

**Authors:** Suzy Gallier, Gary Price, Hina Pandya, Gillian McCarmack, Chris James, Bob Ruane, Laura Forty, Benjamin L Crosby, Catherine Atkin, Ralph Evans, Kevin W Dunn, Eliot Marston, Clark Crawford, Martin Levermore, Shekha Modhwadia, John Attwood, Stephen Perks, Rima Doal, Georgios Gkoutos, Richard Dormer, Andy Rosser, Hilary Fanning, Elizabeth Sapey

**Affiliations:** 1 PIONEER Health Data Research Hub, University Hospitals Birmingham NHS Foundation Trust, Birmingham, UK; 2 PIONEER Data Hub, University of Birmingham College of Medical and Dental Sciences, Birmingham, UK; 3 PIONEER Data Hub, University Hospitals Birmingham NHS Foundation Trust, Birmingham, UK; 4 HDR-UK Midlands Physical Site, University Hospitals Birmingham NHS Foundation Trust, Birmingham, UK; 5 Research Support Services, University of Birmingham College of Medical and Dental Sciences, Birmingham, UK; 6 Research and Development Governance, University Hospitals Birmingham NHS Foundation Trust, Birmingham, UK; 7 Medical Devices Technology International Limited (MDTi), Wolverhampton, West Midlands, UK; 8 Faculty of Business, Law and Social Sciences, Birmingham City University, Birmingham, UK; 9 Health Informatics, University Hospitals Birmingham NHS Foundation Trust, Birmingham, UK; 10 PIONEER Health Informatics, University Hospitals Birmingham NHS Foundation Trust, Birmingham, UK; 11 Institute of Cancer and Genomic Sciences, University of Birmingham College of Medical and Dental Sciences, Birmingham, UK; 12 Insignia Medical Systems Limited, Basingstoke, Hampshire, UK; 13 West Midlands Ambulance Service NHS Foundation Trust, Brierley Hill, West Midlands, UK; 14 Research and Development, University Hospitals Birmingham NHS Foundation Trust, Birmingham, UK; 15 Acute Medicine, Birmingham Acute Care Research, University Hospitals Birmingham NHS Foundation Trust, Birmingham, UK

**Keywords:** information management, health care, record systems, information systems, medical informatics

## Abstract

**Introduction:**

Health Data Research UK designated seven UK-based Hubs to facilitate health data use for research. PIONEER is the Hub in Acute Care. PIONEER delivered workshops where patients/public citizens agreed key principles to guide access to unconsented, anonymised, routinely collected health data. These were used to inform the protocol.

**Methods:**

This paper describes the PIONEER infrastructure and data access processes. PIONEER is a research database and analytical environment that links routinely collected health data across community, ambulance and hospital healthcare providers. PIONEER aims ultimately to improve patient health and care, by making health data discoverable and accessible for research by National Health Service, academic and commercial organisations. The PIONEER protocol incorporates principles identified in the public/patient workshops. This includes all data access requests being reviewed by the Data Trust Committee, a group of public citizens who advise on whether requests should be supported prior to licensed access.

**Ethics and dissemination:**

East Midlands–Derby REC (20/EM/0158): Confidentiality Advisory Group (20/CAG/0084). www.PIONEERdatahub.co.uk

## Introduction

The National Health Service (NHS) is the publicly funded health provider for the UK. Specific NHS organisations hold identifiable patients’ medical information,^1^ increasingly within electronic health records (EHRs). EHRs facilitate health data sharing for personal medical care (the primary purpose of the health data) and also for health service planning, research and innovation, collectively termed ‘secondary uses’.

Health Data Research UK (HDR-UK), the national institute for health data science, coordinated the designation of Health Data Hubs in alignment with the UK’s Industrial Strategy to improve the quality, discoverability and accessibility of health data for research.

PIONEER is a Health Data Research Hub that focuses on providing licensed access to acute care health data. It was developed to curate routinely collected health data from unplanned healthcare contacts across community, ambulance and hospital providers and then facilitate the transparent and ethical use of deidentified data for research and innovation purposes. PIONEER has a principle aim of improving patient care and well-being through research. PIONEER is not a clinical system and does not provide healthcare to individuals. For clarity, PIONEER is not a Health IT System for use in the health and care environment, it sits within the Health and Social Care Research Framework, not driving clinical care for the data subjects within the research database.

‘Acute care’ is any unplanned health episode. Each year the NHS provides approximately 110 million urgent same-day patient contacts^2^ with the numbers rising year on year. There are known health inequalities associated with acute care, as people from lower socioeconomic groups are more likely to present to emergency departments.^3^ Acute care provides a unique and useful microcosm of wider health service challenges, for example, one in five patients with cancer is diagnosed as an emergency, with significant implications for long-term outcomes.^4^

Despite the scale and cost of acute care, this specialty has not benefited from significant innovation.^5^ There is a critical need for new patient pathways, diagnostic processes, therapeutics and devices in acute care. Licensed access to acute care health data could offer unique insights and design potential solutions to some of the challenges this health sector faces.

Previous research suggests there is public support for data sharing but with key concerns.^6^ The PIONEER Hub conducted a series of public and patient events and developed a framework for health data secondary use, in collaboration with patients and public stakeholders.^7^ The agreed framework included health data access if the supported research could benefit patients and citizens, a commitment to transparency with data sharing overseen by the NHS and patient/public involvement in data access decisions. Studies have described greater confidence in data sharing when the NHS is involved,^8 9^ that public involvement is important^10^ and that the principles of data minimisation should be applied.^8^ The PIONEER protocol was developed to ensure this framework was embedded within all operating processes.

## Methods and PIONEER operating processes

### The principle of public good

PIONEER is a partnership between patient and public members, the NHS, universities, data scientists and selected industry partners. For the full protocol, please see the online supplemental file.

10.1136/bmjhci-2020-100294.supp1 Supplementary data

The aim of PIONEER is to link routinely collected health data from acute care providers and use this in an anonymised form to innovate healthcare through research.

PIONEER supports the following objectives:

1. To develop a research database and analytics platform of linked, routinely collected health data from different healthcare providers.
2. To work with patients, the public and other stakeholders to ensure that data access through PIONEER is in the public interest.
3. To make datasets discoverable and appropriately accessible to research organisations, NHS bodies and commercial organisations, where the supported research is likely to lead to patient benefit.
4. To support research, development and innovation within a robust governance system.

The potential uses of the PIONEER dataset include but are not limited to research supporting improvements to service delivery, reducing diagnostic delay, reducing chronic disease burden, and development of new treatments and technologies such as point of care testing, self-management software and live data streaming to provide interventions closer to home and avoid unwanted or unnecessary hospital admissions.

### Proximity to the NHS

University Hospitals Birmingham NHS Foundation Trust (UHB) is the data controller for PIONEER. UHB is one of the largest NHS Trusts in England, with 2750 beds, more than 22 000 staff, an in-house built and clinically lead EHR (Prescribing Information and Communication System) and a shared primary and secondary care record (Your Care Connected).

### Patient inclusion criteria

Any patient with an acute health issue who has sought medical advice/care from a PIONEER data partner. Any and all acute episodes can be included with no restriction on disease, condition or age. Patient data within PIONEER is collected as part of routine care. Longitudinal data will be eligible if it relates to, leads to or stems from an acute care contact. Patients will not need to specifically consent to their health data being used within PIONEER, but patients will not be included if they have chosen to opt out of the use or disclosure of their data for research and planning via the NHS National Data Opt-Out^11^ or if they have opted out of PIONEER specifically (see ethical considerations).

### Included data

PIONEER includes data about patient demographics (age, gender, ethnicity), past and current medical diagnoses, medications, allergies and healthcare contacts. There is serial data on vital signs, investigations including laboratory, pathology, physiology and imaging, all acute medical prescriptions and administrations and health process data (clinical review specialty, ward type). This is supplemented with health data prior to and after the acute care contact, linked across healthcare providers, to enable assessment of preceding health and subsequent outcome. The PIONEER dataset adds to publicly available datasets, as highlighted in the examples provided in table 1.

**Table 1:** A summary of related data sources

Name	Country	Subject areas	Update period	Description
HES	UK	All healthcare	Daily (A&E is quarterly)	High-level, does not include physiological measurements outside of classifications in main diagnoses
MIMIC	USA	Intensive care	Static	Deidentified data covering period '01–'12, in high detail
WHO Global Health Observatory	Global	All healthcare	Variable	High-level count data, on a global scale, does not go to the level of individual patients
Global Health Data Exchange	Global	All healthcare	Variable	Catalogue of existing datasets, generating novel data is outside of its scope
NIHR Health Informatics Collaborative	UK	Thematic		Open only to member of the HIC for collaboration, does not provide a TRE
PIONEER	UK	Acute care including preceding and subsequent health contacts	On demand	Datasets tailored to specific use cases, updated on demand and available via a secure TRE. Individual patient level data that includes medications, physiological measurements, images over 20 years. Individually linked data from primary care, ambulance and secondary care.

This should not be considered fully comprehensive, but highlights the differences between currently available datasets and PIONEER. HES=Hospital Episode Statistics.^24^ MIMIC Critical Care Dataset,^25^ Global Health Observatory,^26^ Global Health Data Exchange^27^ NIHR=National Institute for Health Research Health Informatics Collaborative.^28^

TRE, trusted research environment.

### Research database design and security

Data will be stored on a secure, UHB-controlled Microsoft Azure cloud platform in accordance with the 14 UK Cyber Cloud Principles,^12^ which include data protection in transit; asset protection and resilience; separation between users; a robust governance framework; operational security; secure user management; identity and authentication; and audit. The cloud provision will follow the International Organization for Standardization (ISO) 27 001 standards, an international specification for information security management.^13^ This ISO outlines a broad range of quality control processes, many of which apply to data collection, processing and management with a strong emphasis on information security. An example of how this will be met for PIONEER is that data transferred between organisations will be mathematically checked to ensure it has not been tampered with.

The database platform will comply with the Department of Health Information Governance policies and standards for secure processing of patient healthcare data, as set out in the Information Governance Toolkit of the Health and Social Care Information Centre.^14^ The database platform will undergo cyber-security checks by an independent and external company. The platform’s design enables the rapid build of bespoke trusted research environments (TREs) which provide approved and licensed researchers with access to specific curated, deidentified datasets and a suite of analytical tools (description available from corresponding author). The use of a TRE circumvents data travel from the data controller to the data user. PIONEER is committed to promoting the protection of privacy and data security in line with the Organisation for Economic Co-operation and Development (OECD) Recommendation of the Council on Health Data Governance^15^.

### Data processing

Data is extracted, transformed and securely transmitted to PIONEER/UHB’s managed cloud environment. The data held internally within the PIONEER Research Database is pseudonymised (with personal identifiers replaced with other values (pseudonyms), from which the identities of individuals cannot be intrinsically inferred). Pseudonymisation does not change the status of the data as personal data. Only anonymised data is released to our approved partners (where identifiable data features are removed). The PIONEER Research Database does not contain direct and recognisable identifiers such as name, full address, images of a face or NHS number.

Risk will be managed proportionately when providing access to data that might, alone or through combination, lead to a risk of identification of an individual. A specific example is a postcode. PIONEER holds postcode data to support studies into equity and social deprivation. To reduce risk, a postcode will not be provided directly to researchers. Instead, PIONEER will provide a less specific geographical unit such as the Lower layer Super Output Area (LSOA)^16^ or the associated data of interest such as the Index of Multiple Deprivation score.^17^

Diagnoses including rare diseases are included within PIONEER. A rare diagnosis may enable identification if combined with enough additional indirect identifiers. Access decisions will be evaluated on a case-by-case basis and appropriate restrictions will be placed on accompanying data that might significantly increase the risk of identification. Of note, PIONEER processes were developed after discussion with patients with rare conditions, and they explicitly supported the inclusion of rare diseases in PIONEER (even very rare diseases which risk identification due to rarity) to improve acute care services for these conditions.

### Data quality

Each healthcare provider who uses electronic systems will conduct quality checks on their data to ensure that it is suitable to provide clinical care (the primary purpose of data collection). Data quality assurance (QA) checks will also take place within PIONEER and to ensure the quality of data contained within datasets and PIONEER works towards meeting particular standards, including to the metadata catalogue (ISO 11179),^18^ data quality (ISO 8000)^19^ and QA (ISO 25012).^20^

#### Metadata catalogue (ISO 11179)

A metadata catalogue is used to help the potential user of a database to understand what it contains. The referenced standard addresses the semantics of data, how data is represented and the registration of the data descriptions. ISO 11179 specifically specifies the kind and quality of metadata necessary to describe data as well as the management and administration of that metadata in a metadata registry. The purpose of this standard is to promote the standard description of data and a common understanding of data across and between organisations. PIONEER works to meet this standard through organising data using data element concepts, data elements, conceptual domains and value domains. The 11 179 standard also provides a way to depict relationships among concepts. We use this feature to represent relationships among data. PIONEER links to the HDR-UK Innovation Gateway, which allows researchers to explore datasets, tools, papers and related resources used in health research across the UK through a metadata catalogue, which includes many of the features described above (see https://www.healthdatagateway.org).

#### Data quality (ISO 8000)

Data quality is a broad term that encompasses a range of evaluation and data management techniques. Generally, to be of high-quality data must be considered to be accurate, complete, fit for the required purpose (meeting the needs of the end user) and assessable via a good quality management framework such as those described in ISO 8000. An example of how this will be met for PIONEER is that each table will be inspected to identify fields that are intended to link with other data, and these links will be tested.

#### Quality assurance (ISO 25012)

‘Quality assurance’ is the overarching term for a range of steps that are designed to ensure that a process meets a quality goal; this is distinct from ‘quality control’ that relates to testing the outputs of a QA process. The data quality management framework defines the activities to be undertaken to ensure that data is collected accurately and completely, meeting predefined quality targets and governance constraints. PIONEER data officers will be trained in current information governance good practice and work to a predefined standard operating procedure to meet this standard.

PIONEER improves data quality through checks applied during the processing stage, examples are to identify common data errors such as values that exceed the prescribed limit of characters, to ensure the values are in line and format with those in the NHS Data Dictionary and to ensure all mandatory data fields are complete, including completeness and count checks on the number of NULL values.

If any issues are identified, there is an iterative support loop in place to assist with feeding back and working through if it is a system or user error. The data is then re-extracted and the process of checking resumes until it is ready to stage.

#### Data deidentification

**Figure 1:**
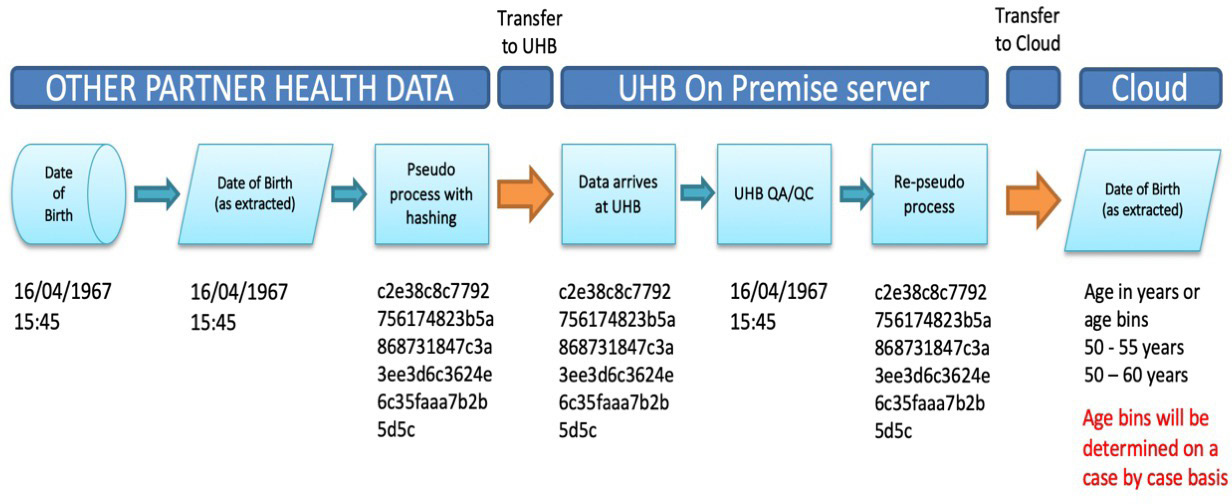
The process of pseudonymising health data in PIONEER. This describes the process to move data using a salt code. Data partners contain identifiable health data, as shown by the date and time of birth. This is extracted from the record in an identifiable form, and then made pseudonymised using a salt code. The date and time of birth cannot be calculated from this hash. The data is then transferred by secure and encrypted pathways to University Hospitals Birmingham NHS Foundation Trust (UHB). At UHB, a proportion of records will have the data made reidentifiable, for QA/QC purposes, but it then remains in the hashed format. If that data is requested, the hash will be transformed into an age in years or into appropriate age bins, as determined on a case by case basis, and as approved by the Data Trust Committee (DTC).

Pseudonymisation is needed for linkage purposes. As PIONEER processes healthcare data, industry standard best practices have been adopted, which ensures compliance with the Information Commissioner’s Office and UK GDPR. These techniques are field level encryption (where the value is replaced with an encrypted version with a key) and hashing. See figure 1 for a diagram of this process. Hashing is a technique that uses standard functions based on mathematical algorithms to create new values. The selected value is represented by a 32-character hexadecimal string (combined letters and numbers), to enhance this security a random ‘salt’ is added. The data are then transferred to the PIONEER database via secure file transfer. Once received by PIONEER the data are reidentified to enable linkage (where applicable) and for QA and quality control checks, before being re-pseudonymised and moved to a secure safe-haven on Microsoft Azure for final processing. Prior to staging for research purposes, data is anonymised and K-anonymity modelling^21^ is performed, to objectively assess the potential for reidentification. The acceptable value will be assessed case by case with data controller and public oversight and agreement.

**Figure 2:**
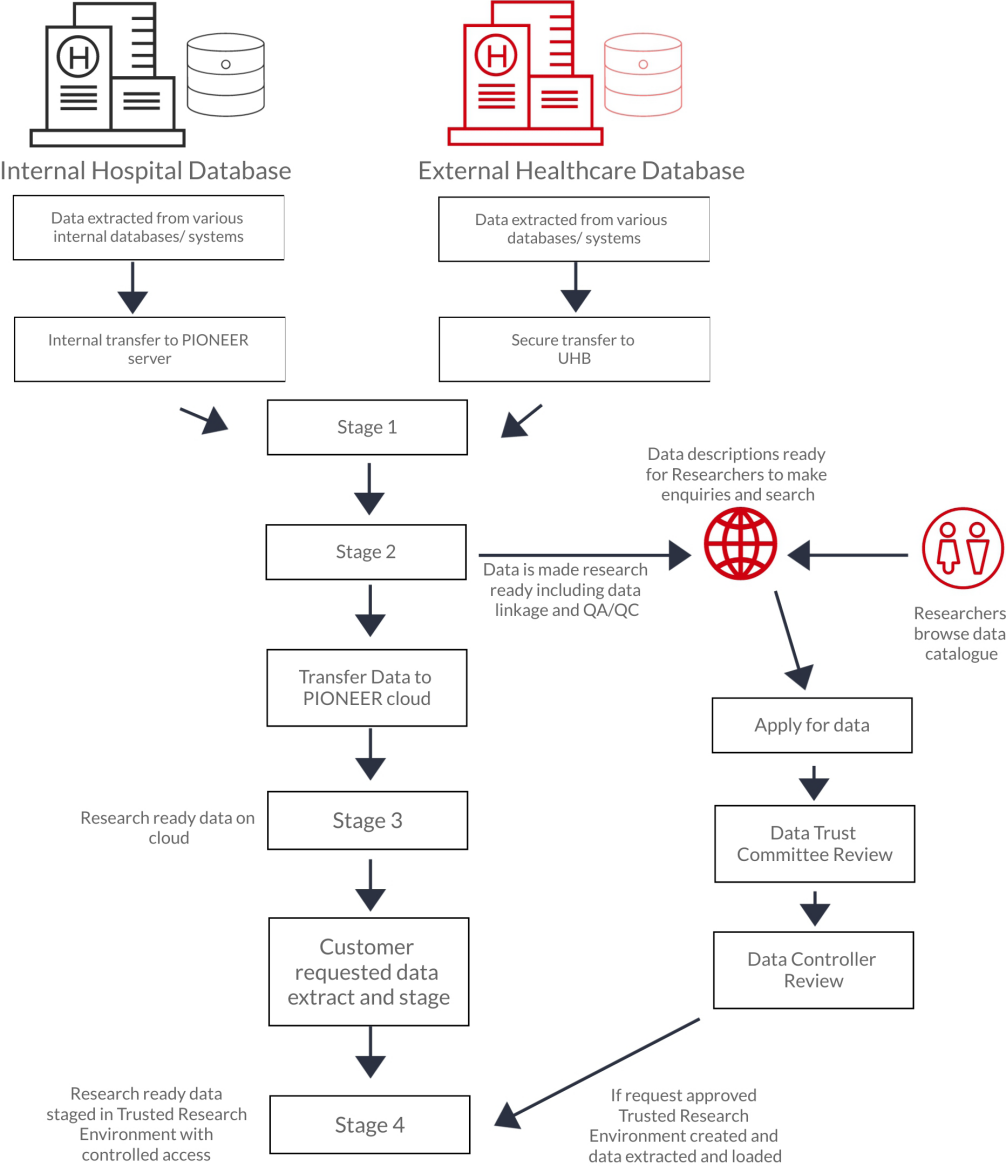
The data staging and access process. Data can enter PIONEER from internal (UHB) and external data providers. At stage 1, data has been cleansed, normalised and sent to UHB in a pseudonymised form. At stage 2, the pseudonymised data is checked, cleansed and undergoes QA/QC by the PIONEER team. From this data, a metadata catalogue is formed, providing high-level data descriptions, which describe the kind of data PIONEER holds. The metadata catalogue is available for researchers to browse. At stage 3, the pseudonymised data is moved to the secure cloud, only accessible by PIONEER team members for further QA/QC processes. If a data request is received, it is reviewed by the Data Trust Committee (DTC) and PIONEER team. The PIONEER team perform a due diligence check and assess the request in terms of risk (see table 1). If the DTC do not support data access, no data access occurs. If the DTC support data access, a data licensing agreement is formed and the exact cut of data required is anonymised, extracted and staged in a bespoke trusted research environment (TRE). At stage 4, this staged and anonymised data can then be accessed by the researchers using specific log on processes and only approved data can leave the TRE (which would be aggregate data and not individual data lines).

Figure 2 is a description of the data preparation process. Data made accessible via PIONEER will be necessary and proportionate to the purposes required, ensuring data minimisation. Data minimisation is assessed on a case by case basis. Data fields shared are only those deemed relevant to the specific research question and limited to what is necessary for the purposes for which they are processed as part of that research question. PIONEER can offer clinical expert consultancy to support this process. Approaches taken include to coarsening some potentially identifiable data (such as age into age groups) or to move a date to the start of a week and adjust all date and time-stamped data accordingly. A risk-based approach to deidentification was adopted to assess the required level of protection, while ensuring the utility and scalability levels required were considered. The data controller maintains the overarching decision to approve the technique selected.

Deidentification of image files involves multiple steps for the three areas requiring attention—image filename; image metadata and identifiers burned onto the image. The image filename is checked to ensure it does not contain any patient identifiers, which are linkable outside the curating organisation, for example, the patient’s NHS number would be replaced with a new unique key value associated with the patient’s clinical data. The image metadata is examined, depending on the type of image (tags for the DICOM standard or EXIF fields for JPG images) to ensure they retain essential clinical information, such as modality, but do not contain any patient identifiers. Where found, identifiers are either removed or replaced with a new unique key value associated with the patient’s clinical data. Lastly, the image is checked to ensure that it does not contain any burned-in patient identifiers. This is done using a combination of manual visual scanning and an evolving AI algorithm, which is being trained to take over this task. Burned-in patient identifiable data is manually removed by obfuscation, while ensuring that any burned-in clinical data is retained.

### Data access

All requests for licensed data access will be reviewed against the principles of the ‘Five Safes’^22^ by the Data Trust Committee, the PIONEER management team and the PIONEER data controller. The ‘Five Safes’ framework is increasingly adopted by health data providers and includes an assessment of the safety of the project, safety of the researchers requesting access to the data, safety of the data (the risk of disclosure or reidentification) and safety of the data setting. PIONEER has contractual safeguards, with data access licensed to expressly preclude any attempts at reidentification and limit the use of the data to the purposes described within the contract, within a specific time frame. The final ‘Safe’ refers to safe outputs, ensuring the statistical results of data analysis are non-disclosive. PIONEER reserves the right to refuse an application or limit the data fields available, based on concerns around possible identification.

### Data request pathways

This process was developed in collaboration with patient and public contributors.

Data requests will be considered from organisations, companies, researchers, members of the public or any agency or body, referred to as data Requestors.

All requests for licensed access to data will be considered against core principles for data access:

1. Benefit to patients, to the NHS, or society.
2. No due diligence concerns
3. Data requests are ethical, appropriate and are not excessive in the data requested nor include data which has more than remote possibility of being reidentified by other data held by the requestor or in the public domain—that is, data requests which pass the risk evaluation.

Data requestors complete a Data Request Form (DRF). The DRF mandates specific training before health data access. This includes reading the Data Security and Protection Toolkit^23^ and the National Data Guardian’s Review of Data Security, Consent and Opt-Outs^1^ as well as taking an e-learning course for data security training. PIONEER will undertake due diligence checking for all data requestors. Requests are then assigned a Data Request Risk Rating: green for low risk, amber for moderate and red for a failed risk assessment. The rating will be based on the data requested, potential for risk and potential for patient gain. See table 2 for an overview of this process.

**Table 2:** An overview of the Data Request Form and data access considerations

Heading	Requirements
**The Data Request Form**	
**A summary of the data request**	
The project: Technical summary	Project title, aims, scientific rationale and background in technical language.
The project: Lay summary	Project title, aims, scientific rationale and background in lay language.
Patient and public involvement	To describe the patient and public involvement and engagement (PPIE) work completed so far and to offer the opportunity for PIONEER supported PPIE.
Expected value of the project to the NHS and general public	To describe how the project is likely to lead to patient and public benefit.
Data requirements and analysis plan	This includes an exact description of the data fields required, whether aggregate or individual data lines are needed, and the justification for this. PIONEER offers the ability to perform analysis on behalf of the researchers. If the researchers wish to perform their own analysis, a description of techniques and tooling required is requested.
Security	Data access is only permitted with a data licensing agreement, but if necessary, anonymised data can be sent to an external trusted research environment (TRE). The security arrangements for this TRE will be listed and reviewed by the PIONEER team, including whether specific ISO standards are met.
Expertise	Listed to ensure the researchers have the relevant training and expertise to conduct the analysis.
Dissemination plan	PIONEER supports open access of data outputs to ensure insights benefit as many people as possible. The ‘Five Safes’ includes an assessment of whether data outputs could lead to patient reidentification, so the DRF includes a description of how this will be avoided.
**Due Diligence**	
**An assessment of the data requestor, which is completed for all data access requests**	
Human rights violations and significant harm	All data requestors undergo a due diligence assessment to determine evidence of serious human rights violations, arms manufacturing or trade and tobacco industry involvement.
Controversies and data breaches	All data requestors undergo a due diligence assessment to determine evidence of data breaches, falsified scientific reports or involvement in significant controversies including financial irregularities and health and safety fines.
**Risk Assessment**	
**A summary of the potential benefit vs risk of the project, the researcher, the data and the data environment**	
Benefit	Designated as clear benefit to NHS patients or society, potential benefit or no potential benefit.
Data	Designated as data which is aggregated or highly unlikely to or may lead to patient identification or data which has a realistic potential patient identification.
Security	Designated as provides evidence of data security which meet all requirements or meet most requirements with additional support or does not meet data security requirements.
Potential reputational risk to PIONEER	Designated as low, moderate or high based on due diligence check.
Previous dealings	To determine the outcome of previous data access activities.
Overall assessment of risk	Low, moderate or high with a recommendation to support or not support data access.

The DRF, due diligence and risk register will be reviewed by the PIONEER senior team, data controller and Data Trust Committee. The specific cut of data required for the project is deidentified and moved to the PIONEER TRE. The data requestor receives specific permissions to access that data within the terms of the Data License Agreement.

### The Data Trust Committee

The Data Trust Committee (DTC) is an advisory function for PIONEER and acts as the public conscience of PIONEER, advising on data release decisions. A decision to support licensed data access by the DTC will lead to data access as long as contractual safeguards are in place. A decision not to support licensed data access by the DTC will be binding for PIONEER and no data will be shared.

The DTC will be made up of individuals recruited by application. All members of the DTC must declare all relevant conflicts of interest. The DTC will be assisted, when needed, by experts in relevant healthcare specialities, data research, information governance and UK data law. These experts will have an advisory capacity only and will not be voting members of the DTC. All data requests will be regarded as confidential. The DTC will discuss each data request form, due diligence and risk assessment and form a consensus decision whether to support the data request. The DTC will seek to form a unanimous decision regarding data access through discussion and reflection. Where this is not possible, non-unanimous decisions to support data access will require 80% of the DTC to support data access. A quorum of at least half of the DTC (rounded up) is required for the DTC to convene and the DTC will generate lay summaries of their activity for public review.

### Building a framework for proportionate, retrospective data release reviews

In time, it is envisaged that the DTC will form proportionate review criteria, categorising some reviews as low risk based on self-developed and tested procedures, which might allow retrospective reviews of some data requests. However, this will only occur when the DTC have been operating for sufficient time to form a view about what a low risk application is, and after specific ethical approval.

### Ethical considerations and opting out

PIONEER operates within Regulation 5 of the Health Service (Control of Patient Information) Regulations 2002 (’section 251 support’) to process confidential patient information without consent. The rationale for using health data without consent was that, to be effective, PIONEER will include data from millions of patients and be fully representative of the patient population as a whole. PIONEER will include data from people who have died and data from people who may not have the capacity to consent, so that the acute health journeys of more vulnerable people also have the potential to benefit from innovation.

As this is an important consideration, the use of data without explicit consent was specifically discussed with more than 300 members of the public prior to the development of PIONEER,^7^ to specifically test if the majority of the public would support data use in this way.

### Transparency

PIONEER is committed to open and transparent communications, which will support and acknowledge patient and public input and help maximise access for high-quality collaborative research, including an Open Science approach and open access publication. PIONEER will provide data in the public domain regarding its operation and purpose.

## Conclusion

This protocol paper outlines the working operations of PIONEER, the Health Data Research Hub in acute care. The ethically approved protocol has been devised to ensure patient and public voices are central in data access decisions and that PIONEER processes reflect the wishes and concerns of patient and public stakeholders. For more information about PIONEER projects and outputs, see www.PIONEERdatahub.co.uk.
