# Supplementary material for: Infrastructure and operating processes of PIONEER, the HDR-UK Data Hub in Acute Care and the workings of the Data Trust Committee: a protocol paper
Source: BMJ Health Care Inform. 2021 Apr 13;28(1):e100294. doi: 10.1136/bmjhci-2020-100294 (PMC8051388; doi:10.1136/bmjhci-2020-100294)
Supplement: Supplementary data [file bmjhci-2020-100294supp001.pdf]

# PIONEER: The UK Health Data Research Hub for Acute Care

# PIONEER

| | |
|---|---|
| Running Title: | PIONEER |
| Protocol Version: | 1.1 |
| Date: | 04/06/2020 |
| IRAS Reference Number: | 279353 |
| REC reference: | 20/EM/0158 |
| Management Reference: | 201911 |
| Funder: | HDRUK |



Gallier S, *et al. BMJ Health Care Inform* 2021; 28:e100294. doi: 10.1136/bmjhci-2020-100294

## CONTACTS

| Role | Contact |
|---|---|
| **Data Controller** | **University Hospitals Birmingham (UHB) NHS Foundation Trust)** |
| **Hub Director** | **Dr Elizabeth Sapey**<br>Honorary Consultant in Acute and Respiratory Medicine<br>Research Lead for Acute Care,<br>UHB NHS Foundation Trust.<br>Email: e.sapey@bham.ac.uk |
| **Information Asset owner, Data Custodian and Information Guardian for PIONEER Research Database** | **Dr Clark Crawford**<br>Head of Research Governance at UHB NHS Foundation Trust<br>Email: Clark.Crawford@uhb.nhs.uk |
| **Information Asset Administrator** | **Suzy Gallier - Clinical Business Lead and Technical Director**<br>**Email: Suzy.Gallier@uhb.nhs.uk** |
| **Patient and Public Involvement (PPI)Lead and Co-investigator** | **Mr Gary Price**<br>Gary.price@eriks.co.uk |
| **Database Project Leads** | **Suzy Gallier - Clinical Business Lead**<br>**Email: Suzy.Gallier@uhb.nhs.uk**<br><br>**Stephen Perks - Principal Developer**<br>**Email: Stephen.Perks@uhb.nhs.uk**<br><br>**John Attwood - Project Manager**<br>**Email: John.Attwood@uhb.nhs.uk** |
| **Funding Body**<br>HDRUK/UKRI | Industrial Strategy Challenge Fund Health Data Research Hub |

## Protocol Approval

### Hub Director:

**Dr Elizabeth Sapey**
Honorary Consultant in Acute and Respiratory Medicine
Research Lead for Acute Care,
UHB NHS Foundation Trust.
Email: e.sapey@bham.ac.uk

Signature:

Date:



Gallier S, *et al. BMJ Health Care Inform* 2021; 28:e100294. doi: 10.1136/bmjhci-2020-100294

# Table of Contents

**PIONEER: THE UK HEALTH DATA RESEARCH HUB FOR ACUTE CARE** ....... **1**

**CONTACTS** ....... **2**

**PROTOCOL APPROVAL** ....... **2**

HUB DIRECTOR: ....... 2

**TABLE OF CONTENTS** ....... **3**

**ABBREVIATIONS** ....... **5**

**TABLE 1: DATABASE RESOURCE** ....... **6**

**1.0 INTRODUCTION** ....... **7**

1.1 DEFINITION ....... 7
1.2 EPIDEMIOLOGY ....... 7
1.3 THE FRAGMENTED NATURE OF ACUTE CARE PROVISION ....... 7
FIGURE 1. OPTIONS FOR ACUTE CARE PROVISION ACROSS THE UK HEALTHCARE SYSTEM ....... 8
1.4 THE CHALLENGE OF ACUTE CARE PROVISION ....... 8

**2.0 PIONEER** ....... **9**

2.1 PIONEER RATIONALE ....... 9
***2.1.1 The West Midlands as a central heart for PIONEER*** ....... *10*
2.2 AIMS ....... 11
2.3 OBJECTIVES ....... 11
2.4 PIONEER DESIGN ....... 12
2.5 PATIENT AND PUBLIC ENGAGEMENT AND INVOLVEMENT IN THE DEVELOPMENT OF PIONEER ....... 14
2.6 TRANSPARENCY IN PIONEER OPERATIONS ....... 15
***2.6.1 Privacy notices provided by the data controller and data collection centres*** ....... *15*
***2.6.2 PIONEER web page*** ....... *16*
***2.6.3 Enquiry forms and email enquiries*** ....... *16*
***2.6.4 Lay summaries, blogs and updates*** ....... *16*
ADDITIONALLY, PIONEER WILL REQUIRE APPLICANTS TO PROVIDE A RESULTS SUMMARY AS A CONDITION OF DATA SUPPLY. THIS INFORMATION WILL BE PUBLISHED ON THE PIONEER WEBSITE UNLESS THE INFORMATION IS AGREED AS COMMERCIALLY SENSITIVE AND UNDER EMBARGO. ....... 16
***2.6.5 Record of applications to PIONEER*** ....... *17*
2.7. PIONEER POPULATION ....... 17
2.8 MAIN INCLUSION CRITERIA ....... 17
2.9 MAIN EXCLUSION CRITERIA ....... 17
2.10 IDENTIFYING POTENTIAL PARTICIPANTS ....... 17
2.11 PIONEER PATIENT DATA PROCESS ....... 17
***2.11.1 Processing patient identifiable data without explicit written consent*** ....... *17*
***2.11.2 UHB NHS Foundation Trust (UHB Data Controllers). Example A1 on figure.*** ....... *19*
***2.11.3 Example A2 (Non-UHB Health Data Partner with an ability to pseudonymise data)*** ....... *19*
***2.11.4 Example A (Non-UHB Health Data Partner without the ability to pseudonymise data)*** ....... *20*
FIGURE 2: PIONEER DATAFLOW PROCESS ....... 21
***2.11.5 Data sensitivity*** ....... *22*
***2.11.6 Process of pseudonymisation*** ....... *23*
***2.11.7 Metadata catalogue*** ....... *23*
***2.11.8. Process of anonymisation and applicant access.*** ....... *24*
***2.11.9 National Data Opt-Out Process*** ....... *24*
***2.11.10. Freedom of Information Act Principles*** ....... *25*

**3.0 PIONEER SCHEDULE** ....... **26**

3.1 MILESTONE 1: HUB PROCESSES ESTABLISHED AFTER PATIENT AND PUBLIC CONSULTATION ....... 26
3.2 MILESTONE 2: SERVICE DELIVERY AND SUSTAINABILITY ....... 26

**4.0 DATA MANAGEMENT** ....... **27**

4.1 DATA COLLECTION ....... 27

**5.0 SOURCE DATA AND DOCUMENTS** ....... **28**



Gallier S, *et al. BMJ Health Care Inform* 2021; 28:e100294. doi: 10.1136/bmjhci-2020-100294

5.1 Data Handling and Record Keeping ........ 28
5.2 Data Validation and quality ........ 28
***5.2.1 Training*** ........ *30*
5.3 Data Security and Access ........ 31
***5.3.1 The 5 Safes: Safe Projects; Is this use of the data appropriate?*** ........ *31*
***5.3.2 The 5 Safes: Safe People; Can the researchers be trusted to use it in an appropriate manner?*** ........ *31*
***5.3.3 The 5 Safes: Safe Data; Is there a disclosure risk in the data itself?*** ........ *32*
***5.3.4 The 5 Safes: Safe Settings; does the access facility limit unauthorised use?*** ........ *32*
***5.3.5 The 5 Safes: Safe Settings; technical security*** ........ *33*
***5.3.6 The 5 Safes: Safe Setting; physical security*** ........ *33*
***5.3.7 The 5 Safes: Safe Settings; network security management*** ........ *34*
***5.3.8. The 5 Safes: Safe Settings; access control*** ........ *34*
***5.3.9 The 5 Safes: Safe settings; contractual safeguards*** ........ *35*
***5.3.10 The 5 Safes: Safe Outputs; are the statistical results non-disclosive?*** ........ *35*
5.4 Database Software ........ 35
5.5 Record Retention ........ 35
5.6 Downstream Security/Integrity ........ 36

**6.0 DATA SHARING ........ 36**

Figure 3: PIONEER Data Access Process ........ 37
6.1 Access to Data Pathway ........ 38
Box 1. PIONEER Data Request Form (indicative content) ........ 39
***6.1.1 Stage One - Technical assessment*** ........ *42*
Box 2. DRF by PIONEER Operations Team ........ 42
***6.1.2. Stage Two – Due Diligence*** ........ *43*
***6.1.3. Stage Two – Further Information*** ........ *44*
***6.1.4. Stage Two – Risk Evaluation*** ........ *44*
Box 3. Data Request Risk Rating ........ 45
6.2 Stage 2: Data Access Decisions and Data Trust Committee Processes ........ 46
***6.2.1 Stage Two – Patient and Public Involvement in PIONEER Data Access Processes: The Data Trust Committee*** ........ *46*
Box 4. Terms of reference for Data Trust Board ........ 49
Figure 4. Data Trust Committee Review Procedures ........ 50
***6.2.2. Data Trust Learning Review*** ........ *50*
6.3 Stage 3. Record and Release ........ 51
***6.3.1 Specific Ethics Committee Approval of Research Projects*** ........ *51*
***6.3.2 Conditions of Data Release to Other Researchers*** ........ *51*
***6.3.3. Data Access Request Denied*** ........ *53*

**7.0 ETHICAL APPROVALS, MANAGEMENT AND GOVERNANCE ........ 54**

7.1 PIONEER Management Committee (PMC) ........ 54
Box 5. Terms of reference for the PIONEER Management Committee ........ 54
7.2 Strategic Executive Group (SEG) ........ 54
7.3. Ethical Conduct ........ 55
7.4. Research Governance ........ 55
7.5 Reporting Breach of PIONEER Policy ........ 55
7.6. Progress Reports and Accountability ........ 56
7.7 Funding and Infrastructure Support ........ 56

**8.0 COMMUNICATION AND DISSEMINATION POLICY ........ 57**

8.1 Communicating and Promoting the Work of PIONEER ........ 57
8.2 Communicating and disseminating research output arising from PIONEER ........ 57

**9.0 ONGOING PPI/E STRATEGY FOR PIONEER ........ 58**

9.1 PPI/E Overarching Aims ........ 58

**10.0 PROTOCOL AMENDMENTS ........ 59**

**11.0 ANNUAL REPORTS AND DISSEMINATION OF FINDINGS ........ 59**

**12.0 REFERENCES ........ 59**

**APPENDIX 1. INNOVATION GATEWAY ........ 61**



Gallier S, *et al. BMJ Health Care Inform* 2021; 28:e100294. doi: 10.1136/bmjhci-2020-100294

**APPENDIX 2. DUE DILIGENCE FORM AND PROCESS ........ 62**

**APPENDIX 3 PPI/E STRATEGY ........ 65**

Defining our principles ........ 66
Defining our stakeholders ........ 66
Defining our existing assets and partners ........ 66
Defining ongoing involvement in PIONEER's structure ........ 67
Defining our approach to informing and refining our long-term PPI/E strategy ........ 67

**KEY AREAS FOR DEVELOPMENT WITH OUR STAKEHOLDERS TO INFORM THE STRATEGY: ........ 68**

Theme 1. Increasing awareness and knowledge ........ 68
Theme 2. Data into action ........ 68
Theme 3. Engagement into involvement ........ 68
Theme 4: Evaluating, sharing and adopting PPI/E best practice ........ 68

## Abbreviations

| | |
|---|---|
| A&E / ED | Accident and Emergency or Emergency Department |
| AI | Artificial Intelligence |
| BORD | Birmingham Out of Hours GP Research Database |
| DB | Database |
| DDF | Due Diligence Form |
| DRF | Data Request Form |
| DSA | Data Sharing Agreement |
| DTC | Data Trust Committee |
| DTLR | Data Trust Learning Review |
| GP | General Practitioner |
| HDRH | Health Data Research Hub |
| HDRUK | Health Data Research UK |
| ITU | Intensive Care Unit |
| NDOO | National Data Opt Out |
| PAS | Patient Administration System |
| PICs | Birmingham Systems Prescribing Information and Communications System |
| PMC | PIONEER Management Committee |
| PPI/E | Patient and Public Involvement and Engagement |
| QA | Quality Assurance |
| REC | Research Ethics Committee |
| RIS | Radiology Information System |
| RWE | Real World Evidence |
| SAMBA | Society Acute Medicine Benchmarking Audit |
| SET | Strategic Executive Team |
| SLA | Service Level Agreement |
| SNOMED | Systematised Nomenclature of Medicine |
| UHB | University Hospitals Birmingham |
| WM | West Midlands |
| WMAS | West Midlands Ambulance Service |



Gallier S, *et al. BMJ Health Care Inform* 2021; 28:e100294. doi: 10.1136/bmjhci-2020-100294

## Table 1: Database Resource

| | |
|---|---|
| Title of database: | PIONEER: The UK Health Data Research Hub in acute care |
| Rationale: | Linkage of routinely collected acute care data from community and secondary care providers to improve unplanned healthcare provision for the UK's population and beyond. |
| Establishment responsible for the database: | University Hospitals Birmingham (UHB) |
| Duration: | 5 years provisionally |
| Resource: | Routine acute care data from healthcare providers |
| Use: | **Generation of a long-term prospective database of linked acute care data to include but not be limited to details of**:<br>Patient demographics<br>The acute care journey and process of care patients undergo<br>The symptoms and cause of the acute care contact<br>Acuity data including measures of how unwell people are on presentation<br>Previous medical and surgical conditions<br>Previous medications and treatments<br>Investigations for the acute presentation including images<br>Treatments provided to the patients<br>Outcomes including escalation of care both within (such as move to intensive care) and outside hospital (such as an increase in social care requirements)<br>**Uses will include the provision of a license to study de-identified patient data in accordance with governance and ethical approval and UK best practice, following contractual agreement with the Data Controller (reviewed by the Data Trust Board) with the specific remit to improve health care and health choices for UK patients within the NHS** |
| Registration: | People with an acute care contact within a data provision partner |
| Inclusion Criteria: | 1. Patients who have sought unplanned or acute health advice or care from UHB or a named data provision partner.<br>2. Patient has chosen to not opt-out. |
| Exclusion Criteria | 1. Patients who have chosen to opt out. |



Gallier S, *et al. BMJ Health Care Inform* 2021; 28:e100294. doi: 10.1136/bmjhci-2020-100294

# 1.0 Introduction

## 1.1 Definition

Acute care is any unplanned health care contact. This can be from a GP but due to a lack of primary care appointments, it is increasingly via out of hours GP services, minor injuries units, hospitals or by calling 111/999. Acute care includes presentations of any cause (medical or surgical, trauma, paediatrics or women's health), and acute care is disease and organ agnostic. In secondary care this includes presentation to the Emergency Department, Acute Medicine, Acute Surgery, and Intensive Care Units. In community services, this can include calling 111 or 999, visiting a pharmacy, seeking an urgent GP appointment, or requesting an urgent, new or increased community service, such as district nurse review or social support to help meet the needs of an unplanned health issue. Increasingly, it is recognised that the care of acutely unwell patients requires specialist skills, with Acute Medicine being recognised as a separate medical specialty since 2009.

## 1.2 Epidemiology

Each year the NHS provides approximately 110 million urgent same-day patient contacts(1), and the number of people seeking unplanned medical help and admission to hospital are rising. The cost of this to the NHS has been estimated at £17bn per year, and frontline NHS staff struggle to meet the demand for patient care. The UK aims to provide A&E care within four hours, however, in recent years the proportion of patients looked after within this target has been falling. This has been caused by rising demand in A&E departments, and an inability to transfer patients to other hospital wards or sites due to delays in the transfer of care from the hospital back to the community(1, 2).

## 1.3 The fragmented nature of acute care provision

Acute care is currently provided by a number of different providers across community and secondary care, as shown in Figure 1.



Gallier S, *et al. BMJ Health Care Inform* 2021; 28:e100294. doi: 10.1136/bmjhci-2020-100294

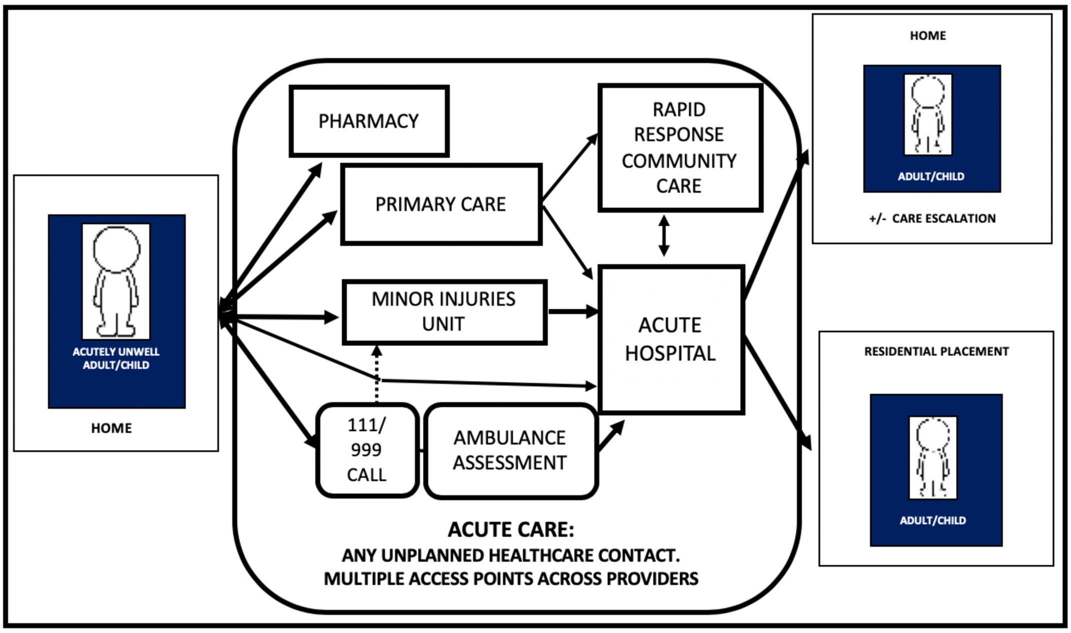


**Figure 1. Options for acute care provision across the UK healthcare system**

Although patients can present to any of these settings, or be transferred between them by ambulance providers, currently little health data is shared between providers. This means healthcare providers are blind to the journeys patients under go as they cross care providers.

These journeys can be convoluted and complex, and the lack of joined up data can hinder the diagnostic process. For example, a real-world journey for one patient consisted of

- a visit to their General practitioner with a lower respiratory tract infection,
- 8 months later, a visit to another GP where a blood test for tiredness identified mild anaemia,
- a trip to an out of hours GP with a urinary tract infection,
- an admission to hospital with sepsis,
- a routine blood test at their GP where mild chronic kidney impairment was noticed,
- a fall and hip fracture treated in a different hospital.

The unifying diagnosis was myeloma and each of these presentations is a recognised feature of the disease, but the diagnosis took 6 years to confirm. A joined-up healthcare system may have been able to identify this disease earlier; a joined up healthcare system with software user prompts to recognise clusters of symptoms could have facilitated this process.

## 1.4 The Challenge of Acute Care Provision

There are known health inequalities associated with acute care and some patients experience poor outcomes. People from lower socioeconomic groups are more likely to present to emergency



Gallier S, *et al. BMJ Health Care Inform* 2021; 28:e100294. doi: 10.1136/bmjhci-2020-100294

departments(3) and, following initial treatment, return afterwards for follow up care(4). Lower socioeconomic status was also associated with poorer outcomes following emergency care even when disease burden was adjusted for(5). Ethnicity also affects the use of emergency care, with those from Black and Minority Ethnic (BME) groups more likely to access care as an acute care contact(6, 7).
One in five patients with cancer are diagnosed as an emergency, which is associated with worse clinical and patient experience outcomes compared with other diagnostic routes; these poorer outcomes are partially but not completely explained by later stage at diagnosis and disease-related complications(8-10). 6.5% of acute presentations relate to adverse drug reactions or side effects from prescribed medications(11). Chronic disease (CD) accounts for two-thirds of emergency medical admissions and approximately 80% of all healthcare costs and the new diagnosis of a chronic disease occurs in 20% of acute care attendees, often at a late stage(12).

Data from the UHB NHS Foundation Trust Emergency Department has shown that in 2018 – 2019, 30% of acute presentations required reassurance without investigation or treatment and 30% required one investigation without admission. 70% of acute care contacts travelled by private car to their care provider, therefore a 30% reduction could save up to 33m car journeys per year. One in three patients with an unplanned admission to UHB had five or more health conditions, but the evidence base for assessing, treating and monitoring multi-morbidity is extremely limited.

There is significant heterogeneity in clinical presentation, burden of symptoms, response to treatment and capacity to recover. However, most acute care guidelines broadly suggest a simple, linear "one size fits all" algorithm to assessment and management, which may not be fit for purpose for today's population.

Despite the scale and cost of acute care, this specialty has not benefited from the same level of innovation or academic endeavour that some other specialties have enjoyed. This was highlighted in the 2018 NICE guidelines for the delivery and care processes within Emergency and Acute Medical Care, where most of the recommendations were expert consensus opinion and great emphasis was placed on the need for further research(13). There is a critical need for new patient pathways, diagnostic processes, therapeutics, and devices in acute care, based on real world evidence, to offer patients the right care at the right time and in the right setting.

## 2.0 PIONEER

### 2.1 PIONEER Rationale



Gallier S, *et al. BMJ Health Care Inform* 2021; 28:e100294. doi: 10.1136/bmjhci-2020-100294

The very scale of the acute care problem could also provide a solution to developing better care for patients. By understanding the acute care experience of patients, there is an opportunity to identify critical points in delivery pathways where new approaches, treatments and devices might revolutionise care. Linking health records across traditionally siloed providers should offer significant benefits to the care of that individual, especially in those patients with complex care needs and multiple health conditions.

With support from patients and the public, linking health records for the population and allowing these anonymised data to be used to understand acute care processes and then model and test new approaches could significantly improve the health care of the nation and help deliver a sustainable NHS. This approach benefits from 'big data' – and the 'bigger' the data, the greater the opportunity.

### 2.1.1 The West Midlands as a central heart for PIONEER

There are strategic advantages for starting this process within the West Midlands (WM). With a population of just under 6 million, the WM has one of Europe's largest, most diverse and non-transient populations. The WM has the highest birth rate in England and is one of the youngest regions with 40% of the population aged under 25. The region faces significant health challenges that impact on regional productivity. Life span is reduced by 1.4 years in females and 1.9 years in males when compared to the South East of the UK. Health span (years spent in good health) is even further reduced with 66.8% of the WM population being obese or overweight. Consequently, citizens experience a high burden of cardiovascular disease, cancer and type 2 diabetes, often at an earlier age than the general population. Poor health drives low socioeconomic status, with the WM having a high percentage gap in employment rates between those with chronic illness, compared to the general population.

It is known that patient's often present to different WM hospitals depending on the nature of their illness, whether individual hospitals are on "divert" or patient preference. However, even hospitals working within the same NHS Foundation Trust (UHB: Queen Elizabeth, Heartlands, Good Hope and Solihull) do not share linked patient records, so these journeys cannot be tracked across centres. Care provided by different NHS Trusts or services are even more siloed, preventing an understanding of how health journeys fit together.

The region also has the tools to drive positive change and improve lives. With forward thinking planners and policy makers, the WM has a well-developed Local Industry Strategy that places health innovation and data science at the heart of regional growth. The WM also includes 18 acute health care trusts, 7 mental health trusts, 1 ambulance service, a thriving MedTech community, and leading academic institutions with a civic focus. This creates significant opportunities through collaborative



Gallier S, *et al. BMJ Health Care Inform* 2021; 28:e100294. doi: 10.1136/bmjhci-2020-100294

working to develop regional innovation and workforce capabilities, which can be scaled-up nationally to improve outcomes for both the local and national populace.

UHB NHS Foundation Trust has developed an award-winning electronic health record. This includes the Birmingham Systems Prescribing Information and Communications System, which is able to capture real time physiological, drug prescribing and administration, investigations and laboratory data, integrated with care processes and patient pathways. This system has been available for over ten years and UHB have significant expertise in implementing regional health data systems as part of clinical care (for example the 100K genome and tertiary referral system for neurosurgery, called the Norse database) within a robust information governance framework. This expertise will be used to implement PIONEER.

PIONEER will gather data from across acute care providers – which will be linked and anonymised within the HDRH - to build the first holistic data-record for acute care. Centring on patient benefit, it will combine routine acute care provision with unparalleled detail and data granularity. UHB will be the data controller for PIONEER and will start by enabling data from patients who present acutely to UHB's four hospitals (Queen Elizabeth Hospital, Heartlands Hospital, Good Hope Hospital and Solihull Hospital) to be linked, so that their care journeys can be understood. Other partners will then join through data sharing agreements, so that these journeys become more completely linked, facilitating the opportunities for improving patient care and choice.

This positions PIONEER as an exemplar for making the NHS 'AI-ready' in an area of critical clinical challenge. Case studies prove the suitability of the acute care environment for artificial intelligence (AI) health applications (e.g. automated prompts to assist with appropriate diagnosis and prescribing(14)). PIONEER will support innovation by making existing inaccessible datasets discoverable, by bringing scale and efficiency to dataset aggregation, and by curation of anonymised routinely collected data.

## 2.2 Aims

The aim of this database is to link routinely collected data from acute care contacts and use this in an anonymised form to innovate health care provision in this hard pressed and challenged sector.

## 2.3 Objectives

PIONEER will support the following objectives:



Gallier S, *et al. BMJ Health Care Inform* 2021; 28:e100294. doi: 10.1136/bmjhci-2020-100294

1. To develop a research database to understand and inform acute healthcare processes and long term consequences for patients admitted to hospital which can inform current and future patient health care and health processes.
2. Work with patients, the public and other stake-holders to ensure that the design, development and governance of data access through PIONEER are in the public interest, and that these principles are communicated effectively on behalf of not only PIONEER, but to improve understanding of the value of health data research and HDR UK more generally.
3. Bring scale and efficiency to dataset aggregation and curation of anonymised routinely collected data relevant to unplanned and acute health care.
4. Make these and existing inaccessible datasets discoverable and appropriately accessible to research organisations, NHS bodies conducting continuous improvement activities (e.g. audit, service evaluation and transformation), and those who are conducting innovation activities which will lead to direct patient benefit.
5. Provide a physical environment of cross-sector collaboration with strong relationships between NHS, industry and academic consortium members to support research, development and innovation.

## 2.4 PIONEER Design

PIONEER is the name of the research database which will collect and link data from national acute care providers.

Initially, the PIONEER Research Database will join acute health data from four hospitals: Queen Elizabeth Hospital, Heartlands, Good Hope and Solihull Hospitals (all part of UHB NHS Foundation Trust). Then this will be linked to the West Midlands Ambulance Service NHS Foundation Trust as a next partner. PIONEER will then identify additional datasets and data collection centres, to provide greater value of the database for research and ultimately patient benefit. Datasets will include but not be limited to health data, as other data sources (patient reported information, pollution measurements) may inform acute care utilisation. All data collection centres will operate within the same mechanism as described below.

Patient data will be collected as part of their routine care when seeking unplanned medical assistance. Initially, any contact with acute care services from UHB (a patient attending the Emergency Department, or acute medical or surgical unit) will be the initial trigger for PIONEER data collection. From that time point, acute care contacts and planned health care utilisation within UHB will be mapped retrospectively and prospectively. This will provide a clear picture of preceding symptoms and health care problems, and prospectively over time, determine changes

in healthcare utilisation after an acute care presentation. This health record will be linked with other acute care contacts from other health data partners within PIONEER for research objectives, such that the patient journey can be tracked, for the first time, across acute health care providers in the West Midlands. Then, all acute care triggers from the WMAS will be included and linked. Ultimately, initiating acute care contacts from other health data providers will also trigger data curation, creating an ever more complete dataset of acute care provision regionally and, in future, nationally. These healthcare contacts are unpredictable, so no minimal or maximal timelines for data acquisition will be set.

PIONEER will be made up of a Director, a Co or deputy director, a Management Team, Workstreams and a coordinating project manager and project officer. This will be referred to as the PIONEER team. This is the operational team.

PIONEER will be guided by the Data Trust Committee (DTC) (see section 6.2.1) – a public and patient group to review and guide all decisions for data release, and a Strategic Executive Group (SEG – see section 7.0)- a group made up of executive personnel from all partners (University of Birmingham, University Hospitals NHS Foundation Trust and other key organisations) to assist with strategic decision making. The DTC is the advisory and the SEG is the strategic support to PIONEER.

The PIONEER team within UHB will lead the design and build of the database (construction configuration, implementation, QA testing) and ensure secure web hosting. The PIONEER team will facilitate data processing for data centres, and provide guide data analysis consultancy whenever these services are needed by applicants to the database.

The potential utility of the PIONEER dataset is vast, with potential benefits including but not limited to improvements to service delivery and design, development of technology, feasibility exercises for clinical trials.

Example potential use cases:

1. Developing and testing self-management software and wearables designed for patients
2. Pathway innovation to tackle diagnostic delay and reduce chronic disease burden
3. Point of care testing and live data streaming to provide interventions closer to home and avoid unwanted or needless admissions
4. New therapeutic targets in drug discovery and real-world trials in acute care
5. Identifying specific populations at risk of poorer outcomes in acute care and those most likely to respond to new therapies



Gallier S, *et al. BMJ Health Care Inform* 2021; 28:e100294. doi: 10.1136/bmjhci-2020-100294

6. Offering more choice in how patients can access the acute healthcare they need when they need it

And to the wider community

1. Up skill the workforce in health data
2. Attract health related industry to the UK
3. Solve our own healthcare challenges
4. Have first access to health innovation across regional providers

To realise these benefits PIONEER will seek to work with the following classes of research bodies:

- NHS Bodies (Trusts, GPs and Health boards)
- Higher Education Institutions (Universities and Colleges)
- Industrial/Commercial Sector – Small, Medium and Large Enterprises
- UK Governmental Bodies
- Charities

## 2.5 Patient and public engagement and involvement in the development of PIONEER

The theme of the PIONEER Acute Care Hub was developed within workshops consisting of 168 members of the public, patients and healthcare providers. We held three separate workshops; one for patients with chronic illness (who were frequent healthcare "users") and their carers; one for members of the public who had not accessed secondary healthcare frequently; and one for NHS hospital staff and GPs. They were asked to consider which parts of healthcare provision needed the most improvement.

The workshops identified that:

1. Unplanned healthcare contacts are the most negative experience within the NHS, noting the lack of new approaches and delays in acute care due to over-stretched front door services.
2. Research needed to be more inclusive: single chronic disease focused research was less able to address the health concerns of our ageing, multi-morbid patients.
3. Research needed to be more inclusive across regional sites, to understand and improve acute healthcare in geographical areas of the greatest need.
4. Research should benefit all ages, including children and older adults
5. Improvements in acute care was the main priority for health innovation (including new ways of accessing healthcare, admission avoidance, hospital care at home, ambulatory care, tracking patients' own health and new therapeutic approaches).



Gallier S, *et al. BMJ Health Care Inform* 2021; 28:e100294. doi: 10.1136/bmjhci-2020-100294

We asked the same 168 people about their thoughts on health data use. After discussing real world examples of how health data had improved aspects of care, 99% of participants were happy for their de-identified health data to be used in research for patient benefit. After discussing real world examples of how health data had improved non-healthcare services (public transport or local services), 96% of participants were happy for their de-identified health data to be used in non-health related research for public benefit. After discussions about the type of researchers who may request access to health data, the principles of GDPR; identifiable data, pseudonymised data and de-identified data, and the principles of appropriate data sharing using the concepts oif the "5 safes", 100% of participants were happy for their de-identified health data to be accessed by NHS staff not directly involved in their care; 98% by academic researchers not involved in the NHS and 94% by industry, if the data would improve health or care for other patients or members of the population.

Since these initial workshops, PIONEER has engaged directly and discussed these issues, including the use of de-identified data without explicit consent, with >300 members of the population. PIONEER involved >40 children aged between 13 and 17 in these discussions, as the national data opt out includes children aged 13 and over.

The results of this PIONEER specific consultation are that the following percentage of patients would be happy for their de-identified health data to be used, without their explicit consent in the following circumstances:

- 98% for research which improves NHS services
- 93% for research undertaken by healthcare staff
- 90% for research undertaken by academic staff not connected to the NHS
- 82% for research undertaken by industry

These initial consultations have informed the design for PIONEER and provided a structure for meaningful PPI/E at the executive heart of PIONEER (see section 9). This consultation process will continue.

## 2.6 Transparency in PIONEER operations

PIONEER will provide data in the public domain regarding its operation and purpose. We will publish this protocol once finalised, as evidence of this.

### 2.6.1 Privacy notices provided by the data controller and data collection centres



Gallier S, *et al. BMJ Health Care Inform* 2021; 28:e100294. doi: 10.1136/bmjhci-2020-100294

The controller and data providers will provide information through their research privacy notices. These notices have been reviewed by PPIE groups and deemed as sufficient and transparent descriptions of the database's intent and operation, and are publicly available for review.
The controller's privacy notices may be found at:
https://www.uhb.nhs.uk/privacy-notice

### 2.6.2 PIONEER web page

PIONEER has a public-facing webpage on the main HDRUK website alongside the other health data research hubs (https://www.hdruk.ac.uk/infrastructure/the-hubs/pioneer/).

The nature and purpose of PIONEER is provided through text but also an introductory video from the leadership team representing the NHS partners and a number of other stakeholders. This is also available at https://youtu.be/tmbHROqNLLA

### 2.6.3 Enquiry forms and email enquiries

Electronic enquiries from the public or potential users can be submitted via the contact forms; in addition, contact details including postal addresses and email is provided:
MDS-c-pioneer@adf.bham.ac.uk

### 2.6.4 Lay summaries, blogs and updates

A condition of all applications for licensed use of data is the provision of a lay summary both of the data request and any outputs. If the application is successful, this lay summary will be published on the PIONEER website after scrutiny by the PIONEER team and the PIONEER Data Trust Committee, to ensure that it is a readily understandable and accurate representation of the project. These will form case summaries of use. Companies will not be able to embargo the lay summary, but commercial sensitivities will be respected by allowing generic summaries to be submitted, and up to a six-month delay between data provision and lay summary release.
Additionally, Pioneer will require applicants to provide a results summary as a condition of data supply. This information will be published on the Pioneer website unless the information is agreed as commercially sensitive and under embargo.

There will also be transparency in the process for evaluating research applications and consideration of data access and use. This includes the standard criteria by which applications are assessed; including public good, the "5 safes" and open access policies. See below in section 5.0 and 6.0.



Gallier S, *et al. BMJ Health Care Inform* 2021; 28:e100294. doi: 10.1136/bmjhci-2020-100294

### 2.6.5 Record of applications to PIONEER

A list of all applications to PIONEER will be available on request, updated on a six-monthly basis. The summary for each application will include the lay summary and the outcome of the application (including any conditions, recommendations, or grounds for refusal). This list will form part of the annual report the PIONEER team will provide to the ethics committee on the date of the favourable ethical opinion.

## 2.7. PIONEER Population

Patients who have undergone an acute care contact, within UHB NHS Foundation Trust or within a health data partner (which could be an NHS Trust, primary care practice, community health service provider or pharmacy, for example). Since there is a critical need for acute health care innovation which is ageless in approach, there will be no upper or lower age limit for data inclusion.

## 2.8 Main Inclusion Criteria

1. Acute care contact within UHB or health data partners.
2. Patient has chosen to not opt-out of the use or disclosure of their data for research and planning.

## 2.9 Main Exclusion Criteria

1. Patients who have chosen to opt out of the use or disclosure of their data for research and planning.

## 2.10 Identifying Potential Participants

Patients with an acute health care contact, initially instigated at UHB NHS Foundation Trust or from any acute healthcare partner or health data partner. Each health data partner will be responsible for patient identification from their own acute care records.

## 2.11 PIONEER Patient Data Process

All steps referred to within 2.9 are presented in Figure 2 – the PIONEER Dataflow Process, where examples of 3 data flows are given. For each example, the steps refer to the numbers in yellow circles within the data flow diagram.

### 2.11.1 Processing patient identifiable data without explicit written consent

Instead of obtaining explicit written consent from each individual patient, section 251 approval is being applied for. The rationale for this is that we wish to:

- Include as many people as possible, with an aim. We need to include all patients who have had an acute care contact within the West Midlands (Connected population clear sight of

6.2M people initially) and then across the UK. We aim to include international datasets, so we can benchmark NHS services and outcomes against the best and worst performing sites internationally, to learn where our services can be improved. UHB alone provides >2.2M care contacts each year and the ambulance service responds to >1.5M calls each year. Including these numbers is vital to allow an in-depth study of acute care across the region, which can provide national and international insight into acute care challenges.

- Link healthcare journeys from the onset of symptoms across primary and secondary care providers. This is to gain an insight into where common delays occur, or where new healthcare services may have prevented an acute presentation, diagnosed a disease earlier or prevented a complication of a chronic illness.
- Include a population that is fully representative of the patient population as a whole, which cannot be achieved from usual research cohorts.
- Include data from people who have died following acute care contacts.
- Include people who may not have the capacity to consent, so that the acute health journeys of more vulnerable adults also have the potential to benefit from innovation.
- Process identifiable data for the purpose of rendering it anonymous at the earliest opportunity.

The scale of the data, and the inclusion of data from people who have died, prevent informed consent being obtained for data use, as would be the usual standard. No additional data to that which is collected as part of standard of care is requested, and all data will be delivered for the research request in a fully anonymised format.

As this is an important consideration, the use of data without explicit consent was specifically discussed with 168 members of the public at a workshop prior to the development of PIONEER, and with >300 people in person, to specifically test if the majority of the public would support data use in this way – see section 2.5 – and this support was given by most.

Patient identifiable data is being processed by the care provider as part of usual healthcare processes and within healthcare governance. The diagram clearly shows where data would then be used by any party for the purposes of research and under the instruction of UHB.

- In marked area A for all sites, data is held on an NHS server. This is identifiable patient data stored for the purposes of health service provision apart from in example A3, where data is sent to UHB when a health data partner who cannot pseudonymise data requires UHB to perform this service.
- In marked area B, data is now pseudonymised but not on the PIONEER cloud.



Gallier S, *et al. BMJ Health Care Inform* 2021; 28:e100294. doi: 10.1136/bmjhci-2020-100294

- In marked area C, pseudonymised data in now placed on the secure PIONEER Microsoft Azure Cloud platform and inaccessible to external researchers. Specific data field can be anonymised here to answer specific research questions.
- Area D is the secure research environment where approved researchers with appropriate data licenses can access the anonymised data staged. This is also where researchers can browse the meta-data catalogue.

### 2.11.2 UHB NHS Foundation Trust (UHB Data Controllers). Example A1 on figure.

Internal data, collected as part of routine clinical care, is pooled from across multiple UHB data systems that hold different types of imaging or other patient centred data (Step 1). Data is then cleansed and linked to ensure quality for clinical purposes (Step 2). The data will be checked in an identifiable form for QA purposes, and any patients who have "opted out" of data sharing will have their record removed (Step 3). The dotted line signifies the start of the research area within the flow sheet and this dotted area signifies where the research protocol begins. Data is pseudonymised using a confidential one-way hash; this will be shared across sites to permit data linkage as patients utilise acute care services across healthcare providers (Step 4), but the data will remain on UHB servers (Step 5). QA checks will ensure the data's accuracy and validity following pseudonymisation (Step 6). Data will remain in the pseudonymised state at all times to allow updated data linkage, as people within the data set have new acute care contacts over time. See freedom of information request principles (section 2.11.9).

Pseudonymised data will be moved to a private and limited access Microsoft Azure UHB cloud (Step 7). Pseudonymised data within this area may be processed for purposes including research, quality improvement projects, audit, and service evaluation by UHB staff under role-based access control, in order to improve UHB hospital services and processes (step 8). This data will then be used to develop the metadata catalogue (Step 9). Here, the data remains until an approved request is received.

### 2.11.3 Example A2 (Non-UHB Health Data Partner with an ability to pseudonymise data)

Internal data is pooled by the health care partner for routine clinical practice as a data controller (Step 1). Data is cleansed and linked as part of routine clinical care, as described above (Step 2). The data will then be checked in an identifiable form for QA purposes, and any patients who have "opted out" of data sharing will have their record removed (Step 3). The dotted line signifies the start of the research area and this is where this research protocol starts. Data is pseudonymised using a secret one-way hash; this will be shared across sites to permit data linkage as patients utilise acute care services across healthcare providers (Step 4). Following pseudonymisation, further QA checks to ensure the data's accuracy and validity are conducted by the data provider, who will still be the data



Gallier S, *et al. BMJ Health Care Inform* 2021; 28:e100294. doi: 10.1136/bmjhci-2020-100294

controller at this point (step 5). Then, pseudonymised data will be provided to UHB (Step 6), and at this point, UHB will become the Data Controller and the healthcare partner will act as the Data Processor. Pseudonymised data will be moved to the private and limited access Microsoft Azure UHB cloud (Step 7). Onward steps are described as above and below in sections 2.11.6 – 2.11.9.

### 2.11.4 Example A (Non-UHB Health Data Partner without the ability to pseudonymise data)

Some potential healthcare data partners lack the digital maturity or staff capacity to be able to pseudonymise their own health data at pace or scale. To enable their participation, and meet a requirement of the PIONEER PPI/E development groups, (that research needed to be more inclusive across regional sites, to understand and improve acute healthcare in geographical areas of greatest need), a third way for data inclusion to PIONEER has been developed, shown in Example A3.

Internal data is pooled by the health care partner, who will be the data controller as per usual clinical practice and as part of routine clinical care (Step 1). From step 1b onwards, data use is for research purposes and the research protocol pathway is initiated. Identifiable data will be sent in a selected and staged manner to UHB. At this point, UHB becomes the data controller and the health care data provider is the Data processor (Step 1b). This is included in the dotted line within the figure, as this represents research activity. Data is then cleansed and linked (Step 2). The data will be checked in an identifiable form for QA purposes, and any patients who have "opted out" of data sharing will have their record removed (Step 3). Data is pseudonymised using a secret one-way hash; this will be shared across sites to permit data linkage as patients utilise acute care services across healthcare providers (Steps 4 and 5). QA checks will ensure the data's accuracy and validity following pseudonymisation (Step 6). Pseudonymised data will then be moved to the private and limited access Microsoft Azure UHB cloud system (Step 7). Onward steps are described as above and below in sections 2.11.6 – 2.11.9.



Gallier S, *et al. BMJ Health Care Inform* 2021; 28:e100294. doi: 10.1136/bmjhci-2020-100294

## Figure 2: PIONEER Dataflow Process

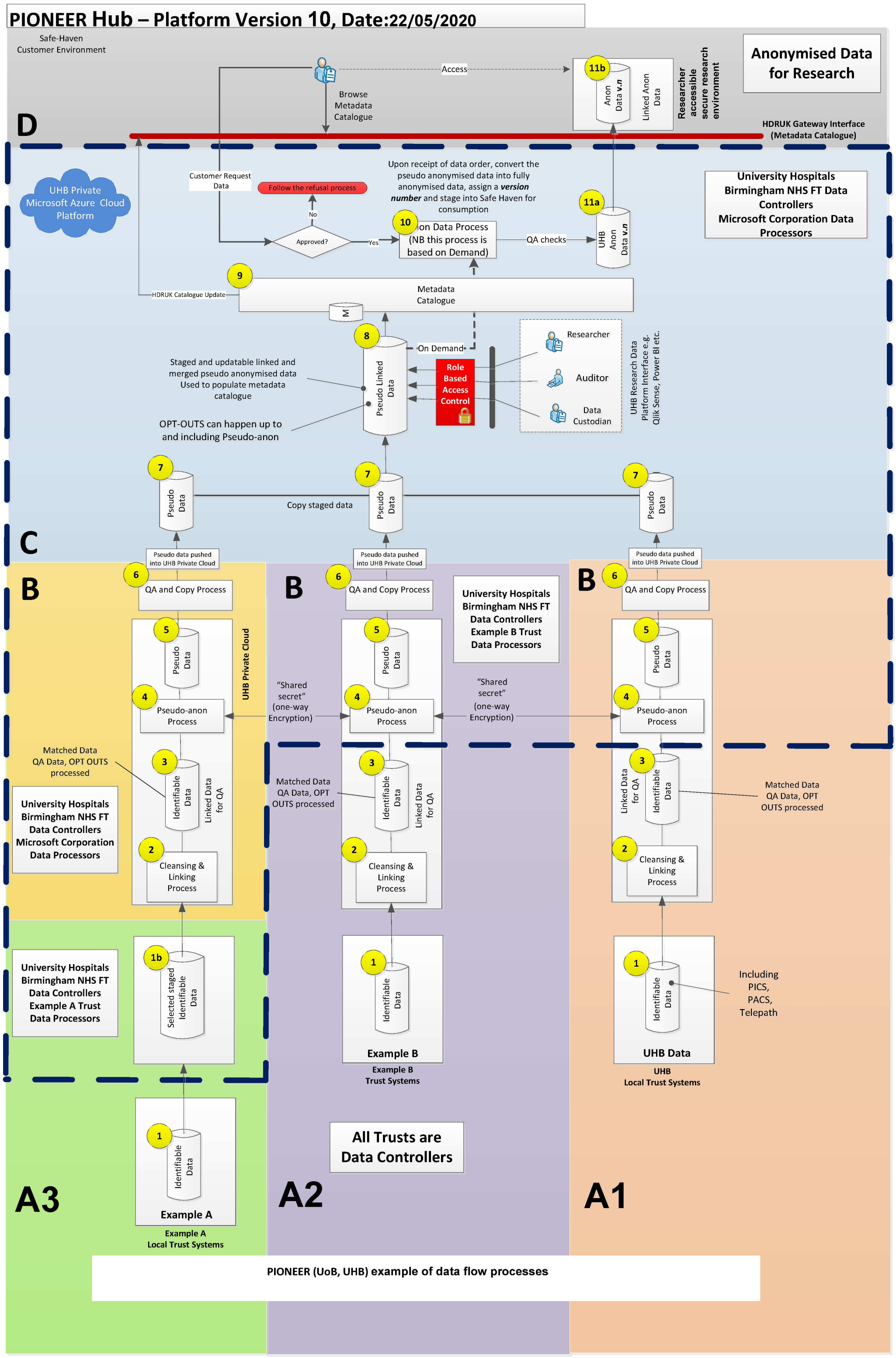




Gallier S, et al. BMJ Health Care Inform 2021; 28:e100294. doi: 10.1136/bmjhci-2020-100294

### 2.11.5 Data sensitivity

*Data sensitivity:* The data within the PIONEER Research Database is data relevant to an individual's systemic health. This includes images of internal structures. A number of highly sensitive types of data relevant to health are excluded; notably data on sexual health and sexual orientation.

*Risk of identification*: The data within the PIONEER Research Database is pseudonymised data, but only anonymised data is released. With regard to class of identification:

Direct identifiers

- The PIONEER Research Database does not contain direct and recognisable identifiers such as name, address or image of a face
- The PIONEER Research Database does not contain direct but not recognisable alphanumeric identifiers such as NHS number
- The PIONEER Research Database does contain images that are not recognisable but may be unique

Indirect identifiers

- The PIONEER Research Database does contain post code, age, gender and diagnoses including rare diseases

Risk will be managed proportionately when providing access to any data that might, alone or through combination, lead to the identification of an individual. Specific examples include:

- Post code: the PIONEER Research Database holds postcode data to support studies into equity of access, and enable greater understanding of the health impacts of social deprivation. To reduce the risk, however, access will not be provided to the postcode directly. Instead, PIONEER will provide the required linked data on demand and provide it as part of the anonymised dataset. For example, providing a less specific geographical unit such as the Lower layer Super Output Area (LSOA), or the associated data of interest such as the Index of Multiple Deprivation score. This approach reduces risk whilst ensuring that the research value of this data is not compromised.
- Age: date of birth is not provided to reduce the likelihood of identification; age is provided to the nearest year.
- Diagnoses including rare diseases: a rare diagnosis may enable identification if combined with enough additional indirect identifiers; this will be evaluated on a case-by-case basis and appropriate restrictions will be placed on accompanying data, such as the specificity of any age or geographical



Gallier S, *et al. BMJ Health Care Inform* 2021; 28:e100294. doi: 10.1136/bmjhci-2020-100294

data provided, that might significantly increase the risk of identification. Of note, PIONEER processes were development after discussion with patents with rare conditions, and they explicitly supported the inclusion of rare diseases in PIONEER (even very rare diseases which risk identification due to rarity) to improve acute care services for these conditions.

- Data in combination: the combination of enough data fields will at some point result in a unique profile for an individual. This provides a theoretical risk to identification, but such identification is still only possible if that same set of data is provided from some other source. Such datasets are not in the public domain, making this risk extremely low.

A general principle of PIONEER is that data made accessible should be necessary and proportionate to the purposes required i.e., there is data minimisation. When requesting access to a dataset, the applicant must justify the inclusion of each data field. PIONEER will review the dataset to be provided to the applicant to satisfy the condition that the risk of re-identification is low. PIONEER reserves the right to refuse an application, or limit the data fields available, based on concerns around possible identification.

### 2.11.6 Process of pseudonymisation

This is a technical process of replacing personal identifiers in a dataset with other values (pseudonyms), from which the identities of individuals cannot be intrinsically inferred. PIONEER maintains an association between the original value and replacement value. Examples of this process are replacing an NHS number with another allocated random number curated within PIONEER. The allocated number has been generated using a specific encrypted 'salt code' added to this, before the combined data is then encrypted using a SHA2-256 hashing algorithm.

Despite this process, the very nature of the linked data across primary care and secondary care providers, even when pseudonymised, means it may not require considerable effort to potentially identify a patient. For this reason, only de-identified data will be shared with external agencies, apart from UHB staff on a rules-based system for audit, quality checks, research, and formation of the metadata catalogue, as outlined in the data flow diagram.

### 2.11.7 Metadata catalogue

A metadata catalogue will be produced, detailing a summary of the data available. This will be refreshed with version control. A copy of the metadata catalogue will be placed on the HDRUK metadata catalogue, and will also be available to browse on the PIONEER webpage. Applicants may browse the HDRUK Metadata catalogue and submit requests for data via the Gateway.



Gallier S, *et al. BMJ Health Care Inform* 2021; 28:e100294. doi: 10.1136/bmjhci-2020-100294

The metadata required by HDRUK is pre-determined and utilises the MoSCoW rating, which seeks to categorise user requests into 'Must have', 'Should have', 'Could have', and 'Won't have'. We are required to provide the 'Must have', and will aim to provide as much of the remaining information as requested, which comprises summary level data.

### 2.11.8. Process of anonymisation and applicant access.

On receipt of an approved request, the requested data will be extracted from the pseudonymised data hub (Step 10) and anonymised (Step 11a). The anonymised data will undergo a QC check for quality and accuracy, and to ensure adequate anonymisation of all data fields. Anonymisation means that information that identifies an individual patient has been removed. The intent of anonymisation is to turn data into a form which does not directly identify individuals, and where re-identification is not likely to take place.

This is a technical process of replacing personal identifiers in a dataset with other values, from which the identities of individuals cannot be obtained. PIONEER does not maintain any association between the original value and the replacement value. Examples of this process are replacing an NHS number with another allocated random number.

Anonymised datasets will be created on demand for specific projects and will be held within the PIONEER data safe haven (11b) for the applicant to access, but this will not be retained and PIONEER will not retain a copy of any anonymised data that was supplied to any user. The code used to create this anonymised dataset will be stored, so that the same dataset could be re-established to check the results of research outputs if needed, but the actual anonymised dataset will be destroyed after use at an agreed date within the data licensed. The pseudonymised data from which the anonymise dataset was made will not be destroyed, and will remain safely within the secure PIONEER cloud.

### 2.11.9 National Data Opt-Out Process

PIONEER will comply with the NHS Digital "National Data Opt-out" (NDOO) policy using the process from NHS Digital outlined below. Data subjects will be informed of the process for 'opting out' via the Trust privacy notice; patients who wish to be excluded can opt out either online or via phone registration. This is recorded on the Spine by NHS Digital, and we will cross check against this prior to data being utilised for research purposes.

The NDOO was introduced to give patients a choice on how their confidential information is used for purposes beyond their individual care. The information that the opt-out applies to is special category data, as it includes information about a patient's health care and/or treatment that has been collected as part of the care provided for the patient. PIONEER follows the NHS Digital's process so that,



Gallier S, et al. BMJ Health Care Inform 2021; 28:e100294. doi: 10.1136/bmjhci-2020-100294

"patients can set or change their national data opt-out choice using an online process or contact centre service". When a patient sets a national data opt-out it is held in a repository on the NHS Spine against the patient's NHS number.

The Data controller for each dataset will be asked to check if any patients have opted-out of data use; however, it is recognised that patients may choose to opt out after their data has entered PIONEER. In accordance with the patient's wishes and the national data opt-out policy, as a health and care organisation located in England, PIONEER is "required to apply national data opt-outs when applicable to a use or disclosure of confidential patient information for purposes other than the patient's care or treatment."

In line with the NHS Digital process, PIONEER will check, by using the NHS numbers of patients, whether a patient has registered to opt-out before the data is used/disclosed. To do this, a separate list of the NHS numbers in the data that will be used/disclosed needs to be created. The list of NHS numbers is then submitted to the Check for NDOO service, via the secure Message Exchange for Social Care and Health (MESH) messaging service. The Check for NDOO service is an external service provided by NHS Digital. The service checks the list of NHS Numbers against a list of opt-outs created from the repository on the NHS Spine. Where a match is found, it removes the NHS number from the list and then returns an updated list of NHS numbers (with opt-outs removed) back to UHB via MESH.

This creates a 'cleaned' set of data with opt-outs applied that PIONEER can then use/disclose. If a patient chooses to opt out after data processing has occurred, then their record will be removed, provided a link to this record still exists i.e. if this is pseudonymised data. The opt out does not apply to fully anonymised data, since at that point there is no link back to the patient from which it derived.

### 2.11.10. Freedom of Information Act Principles

- PIONEER will process any Freedom of Information (FOI) Act requests to meet all requirements.
- Each FOI request will be considered individually.
- Given the in-depth nature of linked data within PIONEER, even following pseudonymisation, it would not take considerable effort to potentially identify an individual from their linked, pseudonymised data, especially were that data linked to external datasets. As PIONEER cannot eliminate the risk of re-identifying the individual with pseudonymisation, it is highly unlikely that the release of pseudonymised data will be permitted under any FOI enquiry.
- All releases of data are for a specified purpose and the use of the data is restricted by conditions specified within the Data Licence Agreement (DLA).



Gallier S, *et al. BMJ Health Care Inform* 2021; 28:e100294. doi: 10.1136/bmjhci-2020-100294

- Any attempt by a receiving organisation to re-identify any patients whose records are provided in anonymised form would be considered a breach of the Data Protection Act and the DSA.
- Anonymised datasets will be created on demand for specific projects and will be held within the PIONEER data safe haven (11b) for the applicant to access, but PIONEER will not retain a copy of any de-identified data that was supplied to any user.

## 3.0 PIONEER Schedule

The target schedule for the setting up of the database is as follows:

### 3.1 Milestone 1: Hub processes established after patient and public consultation

October 2019 – May 2020

- Consortium Agreement in place with all key partners
- Patient and public workshops and questionnaires to establish hub operational processes
- Patient Groups established and PPI/E strategy written
- Protocol written and IRAS/ CAG application developed
- Data platform designed and approved by Data Controllers for security
- Key roles recruited
- Governance boards, reporting, and operational processes established

### 3.2 Milestone 2: Service Delivery and sustainability

June 2020 – March 2021

- Ethics and CAG submission and approvals
- Data platform built and tested
- Health data from UHB site placed on platform
- Service Delivery - Evidence that the quality of the datasets has been improved (curated), and that this curated data is discoverable through the Gateway.
- Provide publishable enhanced service case studies (e.g. from industry, academia and NHS) that demonstrate impact (and expected impact) and value to researchers and innovators from a range of sectors, and to patients and populations.
- Provide evidence that the Hub is continuing to engage and involve patients and the public in a meaningful manner.



Gallier S, *et al. BMJ Health Care Inform* 2021; 28:e100294. doi: 10.1136/bmjhci-2020-100294

Partners and collaborators will be engaged early and often. Relevant partners responsible for implementation will be integral to goal setting and planning for each delivery iteration, to ensure a common understanding and commitment towards targets.

Key performance indicators will be established to meet applicants expectations and deadlines. Furthermore, the Director will liaise with existing and future partners to ensure their needs are seamlessly considered and anticipated. PIONEER will evidence a sustainable business case to support the future service offering of the Hub.

Provide additional evidence that the Hub is engaging and involving patients and the public, and that involvement is at the centre of its governance processes.

For Sustainability and Scalability – PIONEER will evidence enhanced service delivery, demonstrating impact and value to researchers, innovators, patients and populations.

Ongoing costs (beyond year 2) have been projected and a business model has been developed for cost-recovery measures for sustainability and growth.

Potential revenue streams and customers have already been identified. For instance, PIONEER has been incorporated into 2 infrastructure bids and is engaging in early discussions for funded end uses.

## 4.0 Data Management

### 4.1 Data Collection

Data will consist of routine, pre-existing acute care data. This will include demographic data, data of care processes (time acute care presentation, first assessment, first investigations and treatment, time to discharge, grade of staff, place of care), and health care delivery (investigations and treatments, diagnosis and onward care plans). Investigations will include imaging (radiographs, computer tomography, magnetic resonance images etc) as well as physiological data captured as reports and images (such as electrocardiograms and echocardiograms). The images are already stored on the trust local servers in data warehouses within the trust's own legal entity. Within the UHB data warehouse, data is stored from sources such as PAS and Medisoft. See section 2.11.4 for special category data.
Refer to Figure 2 - PIONEER Dataflow Process for the data collection process.



Gallier S, *et al. BMJ Health Care Inform* 2021; 28:e100294. doi: 10.1136/bmjhci-2020-100294

# 5.0 Source Data and Documents

## 5.1 Data Handling and Record Keeping

Data will be submitted directly to a secure UHB owned cloud-based environment, maintained on the Microsoft Azure cloud platform in accordance with the UK Cyber Cloud Principles which are outlined here:

https://www.ncsc.gov.uk/collection/cloud-security?curPage=/collection/cloud-security/implementing-the-cloud-security-principles

The cloud provision will follow the standards below:

**ISO 27001**

An international specification for information security management. The corresponding code of practice is ISO/IEC 27002.

**ISO 27017**

Code of practice for information security controls based on ISO/IEC 27002 for cloud services.

**ISO 27018**

Code of practice for protection of Personally Identifiable Information (PII) in public clouds acting as PII processors.

The database platform will comply with the Department of Health Information Governance policies and standards for secure processing of patient healthcare data, as set out in the Information Governance Toolkit of the Health and Social Care Information Centre.

Access to data on the cloud will be limited to UHB staff, specifically those within the data management teams in UHB informatics. These staff have access to identifiable data as part of their role in the trust to process data for service improvement and healthcare provision.

## 5.2 Data Validation and quality

Data will be cleansed and matched in each trust's local server, as per usual data controllership activities (as described in Figure 2). Data cleansing is the process of detecting and correcting (or removing) corrupt or duplicate or inaccurate records from a record set, table or database. It refers to identifying incomplete, incorrect, inaccurate or irrelevant parts of the data, and then replacing, modifying, or deleting the dirty or coarse data.

Secondly, the data will be normalised; this is the systematic process to ensure the data structure is suitable or serves the purpose. Here, the undesirable characteristics of the data are eliminated



Gallier S, *et al. BMJ Health Care Inform* 2021; 28:e100294. doi: 10.1136/bmjhci-2020-100294

or updated to improve the consistency and the quality. The goal of this process is to reduce redundancy, inaccuracy, and to organise the data. The data will only be pseudonymised when these processes are complete.

Quality Assurance (QA) of the data will take place within the local trust servers prior to transfer to Microsoft Azure Clouds, and during the anonymisation process in the Shared Private Microsoft Cloud. The QA will check whether the record counts are correct as per expectations; whether mandatory fields are populated; whether the primary and foreign keys work; and whether the pseudonymisation has been successful if SNOMED codes have been appended.

The project will require ongoing access to clinical systems, and by doing so it will support maintenance of accurate data. Data will be either refreshed (pulling new accurate information), or cross checking of data will occur on a frequent basis. The published date is a mandatory field in the metadata catalogue and will be clearly identified. Once the data has been anonymised there will be no ability to update the anonymised data sets. However the code pertaining to that version of the anonymised data will be keep so that the same anonymised dataset can be generated. Alternatively, another fully anonymised data set can be produced, and this would be version controlled by the date against which it was produced.

To ensure the quality of data contained within datasets the quality processes below will be used against the datasets

Firstly, the processes will perform against particular “Standards”

- ISO 11179 Metadata standard
- ISO 8000 Data Quality
- ISO 25012 Quality Assurance

Secondly each dataset will be checked for completeness and consistency, such that the data contained within is appropriate for that dataset and the data is accurate and cleansed.
To help achieve the required data quality a 'Plan-Do-Review' process will be used. This will be coupled with the following controls:

- Dataset Version Management
- Access control for curated datasets under version control
- Risk-management Controls
    - o e.g. Security controls
- Role-based access

  - e.g. Manual quality-check 'gateways'

    e.g. 'Sensitive/Personal Information' removed
- Pseudonymisation correct and traceable
- Anonymisation correct and untraceable
- Categorised Reference Data (aka Master Data)
- Categorised Transaction Data
- ASCII character set or Unicode
- Mandatory fields populated
- Range constraint on data fields (e.g. Age 0 to 150)
- Remove leading and trailing non-visible characters
- Primitive Data-type constraint (e.g. integer, decimal, string, Date)
- Entity Data-type constraint (e.g. DoB, Country Code, Disease, Postcode, SNOMED)
- Uniform spelling
- Duplication alerts
- Missing data alerts
- Semantic compatibility / ontology-checked (e.g. NHS & PIONEER data-dictionary)
- Foreign-keys matched to Primary keys within included tables
- Auditability built-in / considered from the start

The aim of the project is to link patient journeys across Acute Care settings. A shared 'secret salt' will enable this process to happen, by facilitating consistent pseudonymisation, such that the same patient would always have the same pseudonymised ID regardless of the Trust undertaking the pseudonymisation.

These processes will help ensure the data quality of the PIONEER data.

Only when these processes are completed will the data be pseudonymised. Quality checks will ensure data quality prior to releasing data onto each of the next stages as shown in figure 2.

### 5.2.1 Training

As this is an innovative project there will be ongoing development to support the application of the data, including but not limited to:

- Data Protection and Information Governance – including institutional GDPR and Cyber security training and Data Security Awareness Programme provided by NHS Digital and Health Education England (see https://www.e-lfh.org.uk/programmes/data-security-awareness/)
- Data dictionary creation

- Support with analysis of the data
- Development and testing of algorithms to improve patient care delivery

## 5.3 Data Security and Access

PIONEER is committed to promoting the protection of privacy and data security in line with the OECD Recommendation of the Council on Health Data Governance, and to use a proportionate approach to the governance of data access based on the five "safes''(15). PIONEER recognises the model's key feature that the five dimensions 'severally and jointly' contribute to the safety (or risk) around data access.

### 5.3.1 The 5 Safes: Safe Projects; Is this use of the data appropriate?

*'Safe projects' refers to the legal, moral and ethical considerations surrounding use of the data.*

One of the essential criteria for all projects requesting access to data will be to demonstrate likelihood of patient benefit. Specifically, the project will be evaluated against:

- Does the research aim to bring patient benefit ('public good'), specifically the patient population represented by the data subjects?
- What is the predicted size of that benefit?
- What is the likelihood of the project being successful and this benefit being realised?
- What is the risk of unintended harms including potential discrimination?

It should be noted that there may also be a risk of 'loss to public benefit' through not doing the project.

### 5.3.2 The 5 Safes: Safe People; Can the researchers be trusted to use it in an appropriate manner?

*'Safe people' reviews the knowledge, skills and incentives of the users to store and use the data appropriately.*

*UHB has a longstanding expertise in managing sensitive healthcare data and is host to a number of Research Databases. The teams involved in the design of the database, the use of cloud storage and routine processing of data have skills, training and experience to do so safely. UHB has taken additional precautions to seek external consultancies and legal advice to verify and confirm the suitability of both the cloud platform and the data processing architecture built within it.*

One of the essential criteria by which PIONEER will evaluate all applications will be whether the applicant is deemed to be appropriate. Specifically, the applicant will be evaluated against:

- Can the applicant be trusted to use the data exclusively for the purpose agreed, and on the terms agreed?
- Does the applicant understand the reasons for the restrictions of use, including restrictions on onward data transfer, linkage or manipulation?
- Do they have the necessary skills to undertake the work described and deliver trustworthy outputs?
- Do they have the resources to complete the project?

Evidence for answering the above questions will be supported by the PIONEER Due Diligence Process (DDP), which is outlined in Appendix 2.

Part of ensuring 'Safe people' is that a condition of access for successfully approved projects is for the applicants to undertake relevant training provided by PIONEER and to engage constructively throughout the life of the project to ensure understanding and active acceptance of access conditions, which will support appropriate safe behaviour.

### 5.3.3 The 5 Safes: Safe Data; Is there a disclosure risk in the data itself?

This is discussed in section 2.11

### 5.3.4 The 5 Safes: Safe Settings; does the access facility limit unauthorised use?

PIONEER provides a safe setting through technical and physical security, education and culture, and contractual safeguards. It is enhanced by high-powered computing services, secure access, analytics, and data exchange support, leveraging proven delivery expertise through UHB and Microsoft.

Access rights to data are limited by dual factor authentication for cloud access. PIONEER password policy will following NCSC guidance (as laid out in https://www.ncsc.gov.uk/section/advice-guidance/all-topics ) with specified role rights. Data will be stored on a central web-based platform that is secured. The platform will be located on a private shared cloud provided by Microsoft Azure. Central data will only be accessible as approved by the Data Controller following a use-based access control for the purpose of audit, QA checks and reports.

The PIONEER system will be installed on the Microsoft Azure platform, and will have the backup and recovery tools provided by Microsoft to protect data and installations.

A comprehensive audit trail is in place for the PIONEER system, and the datasets record these footprints:

- who has accessed the system and when,
- when data items are created and who by,
- when data items are edited and who by,
- when datasets have been browsed, or information (with correct permissions) has been accessed and downloaded

### 5.3.5 The 5 Safes: Safe Settings; technical security

Enhanced by high-powered computing services, secure access, analytics and data exchange support, leveraging proven delivery expertise through UHB and Microsoft.

The PIONEER structure is guided by FAIR Data Principles (findable, accessible, interoperable and reusable). Access and usage of the secure infrastructure will mean implementing the DSP Toolkit and BS-ISO-27000 Series of Information Security Standards.

### 5.3.6 The 5 Safes: Safe Setting; physical security

The database will sit on a secure UHB tenancy on a Microsoft Azure Cloud instance. This Cloud instance will be located in either the UK South or UK West Microsoft data centres. However, should capacity be in question, PIONEER may use Microsoft data centres in Europe and the USA as Microsoft's Privacy Shield registration provides robust security including meeting requirements of GDPR.

The Azure Cloud data centre's physical security features a layered security model, including safeguards like custom-designed electronic access cards, alarms, vehicle access barriers, perimeter fencing, metal detectors, and biometrics, in addition to the data centre floor featuring a laser beam intrusion detection system.

Microsoft data centres are monitored 24/7 by high-resolution interior and exterior cameras that can detect and track intruders.

Access logs, activity records, and camera footage are available in case an incident occurs. Microsoft data centres are routinely patrolled by experienced security guards who have undergone rigorous background checks and training. Access to the data centre floor is only possible via a security corridor which implements multi factor access control using both security badges and biometrics. Only approved employees with specific roles may enter.



Gallier S, *et al. BMJ Health Care Inform* 2021; 28:e100294. doi: 10.1136/bmjhci-2020-100294

Data is broken into subfile “chunks,” which are stored on local disks and are identified by unique chunk IDs. Microsoft encrypts data as it is written to disk with a per-chunk encryption key that is associated with a specific Access Control List (ACL). The ACL helps ensure that data in each chunk is only decrypted by authorised Microsoft employees and services that were given permission at the time of encrypting the data. This means that different chunks are encrypted with different encryption keys, even if they belong to the same applicant.

These chunks are encrypted using 128-bit or stronger Advanced Encryption Standard (AES).

### 5.3.7 The 5 Safes: Safe Settings; network security management

Within UHB the network security will be controlled with the Trust network security protocols. Any data leaving UHB will be encrypted in transit and at rest. Data transfers from organisations contributing datasets will be done via sFTP between servers (secure File Transfer Protocol).
Data stored on Microsoft’s infrastructure is automatically encrypted at rest and distributed for availability and reliability (as above). This helps guard against unauthorised access and service interruptions.

Penetration tests for external-facing systems:
Data on internal UHB systems will be protected by Sophos. The system sits on the secure UHB Informatics tenancy on Microsoft Azure Cloud instance where it will be protected by Azure’s Security Centre. The Security Centre helps safeguard Windows servers and clients with Windows Defender Advanced Threat Protection, and helps protect Linux servers with behavioural analytics. For every attack attempted or carried out, we would receive a detailed report and recommendations for remediation.

The PIONEER system will have been penetration tested by an external ethical hacking company if required. Microsoft themselves utilise Red Teaming, a form of live site penetration testing, against Microsoft managed infrastructure, services, and applications. Further penetration testing will continue throughout the duration of the database’s operation.

### 5.3.8. The 5 Safes: Safe Settings; access control

Technical authorisation/access will include specific access points via two-factor authorisation, combined with a recorded Media Access Control (MAC) address. Azure Databricks caters for integration with the Azure Active Directory, supporting two-factor authentication, and secure, encrypted transport layers.



Gallier S, *et al. BMJ Health Care Inform* 2021; 28:e100294. doi: 10.1136/bmjhci-2020-100294

### 5.3.9 The 5 Safes: Safe settings; contractual safeguards

Access to data will include contractual obligations which:

- expressly preclude any attempts at re-identification
- limit the use of the data to the purposes described within the contract
- require clients to seek approval from the database before transfer to a third party and to "flow down" all requirements through sub-contracts.
  - Require clients to provide evidence of data destruction.
  - Provide UHB with the right to audit any activity by the client and its subcontractors.

### 5.3.10 The 5 Safes: Safe Outputs; are the statistical results non-disclosive?

It is important that researchers publish their findings, and with sufficient detail to maximise the value of the study. However, the way that data is presented, particularly in tables, may provide sufficient detail for inadvertent disclosure at individual level. Applicants will be required to have considered the risk of re-identification of their requested patient level data. Anonymisation of identifiable data through the removal of direct identifiers will be the first step, the second will be through an 'output statistical disclosure control', in which they evaluate all statistical output for risk of disclosure. A common example is for tables where any cells may have less than five units. In such cases, we would consider either: (1) collapsing categories if possible; or (2) replacing the cell count with '<5'.

## 5.4 Database Software

The software will be compatible with all modern web browsers: i.e. Internet Explorer, Firefox and Safari. The software has a high level of security and encryption. It has multilevel security, data encryption for storing sensitive information, and password protection for data entry and retrieval. Access to the data is controlled through a Roles Based Access control (RBAC).

## 5.5 Record Retention

The application for the PIONEER Research Database is initially for five years, but with the expectation for future applications to maintain its benefits in the long term. Anonymised datasets created on demand will be timestamped and made available under contractual arrangements for pre-specified time periods in line with the nature of the projects.

Requests, reviews and release documentation will be stored for 5 years to allow audit and scrutiny of decision-making procedures. Data on any deviations/breaches may be kept indefinitely to allow for assessments of corrective and preventative actions.



Gallier S, *et al. BMJ Health Care Inform* 2021; 28:e100294. doi: 10.1136/bmjhci-2020-100294

### 5.6 Downstream Security/Integrity

Access to the data under the agreed approval will be on condition of a 'safe setting' for its analysis and use. PIONEER will require assurance of compliance with relevant standards (notably ISO 27001 and the DSP Toolkit), and may request evidence of systems, policies or procedures to ensure such compliance. This will be reflected in data licence agreements.

## 6.0 Data Sharing

Pathways to enable appropriate data sharing have been developed with reference to the principles of the Open Research Concordat (16) and in partnership with patient and public partners. This concordat sets out ten principles with which all those engaged with research should be able to work. These principles are:

1. **Open access to research data is an enabler of high-quality research, a facilitator of innovation and safeguards good research practice**
2. **There are sound reasons why the openness of research data may need to be restricted but any restrictions must be justified and justifiable.**
3. **Open access to research data carries a significant cost, which should be respected by all parties.**
4. **The right of the creators of research data to reasonable first use is recognised.**
5. **Use of others' data should always conform to legal, ethical and regulatory frameworks including appropriate acknowledgement.**
6. **Good data management is fundamental to all stages of the research process and should be established at the outset.**
7. **Data curation is vital to make data useful for others and for long-term preservation of data**
8. **Data supporting publications should be accessible by the publication date and should be in a citeable form.**
9. **Support for the development of appropriate data skills is recognised as a responsibility for all stakeholders.**
10. **Regular reviews of progress towards open research data should be undertaken.**

Pathways to enable appropriate data sharing have been developed in partnership with patient and public partners.

PIONEER is committed to the following principles:

1. Maintaining the highest standards of rigour and integrity in all aspects of research and data access;

2. Ensuring that research which includes PIONEER data is conducted according to appropriate ethical, legal and professional frameworks, obligations, and standards;
3. Supporting a research environment that is underpinned by a culture of integrity and based on good governance, best practice, and support for the development of researchers;
4. Working together to strengthen the integrity of research and to review process for data requests regularly and openly.

Figure 3 provides an overview of the process for data access.

## Figure 3: PIONEER Data Access Process

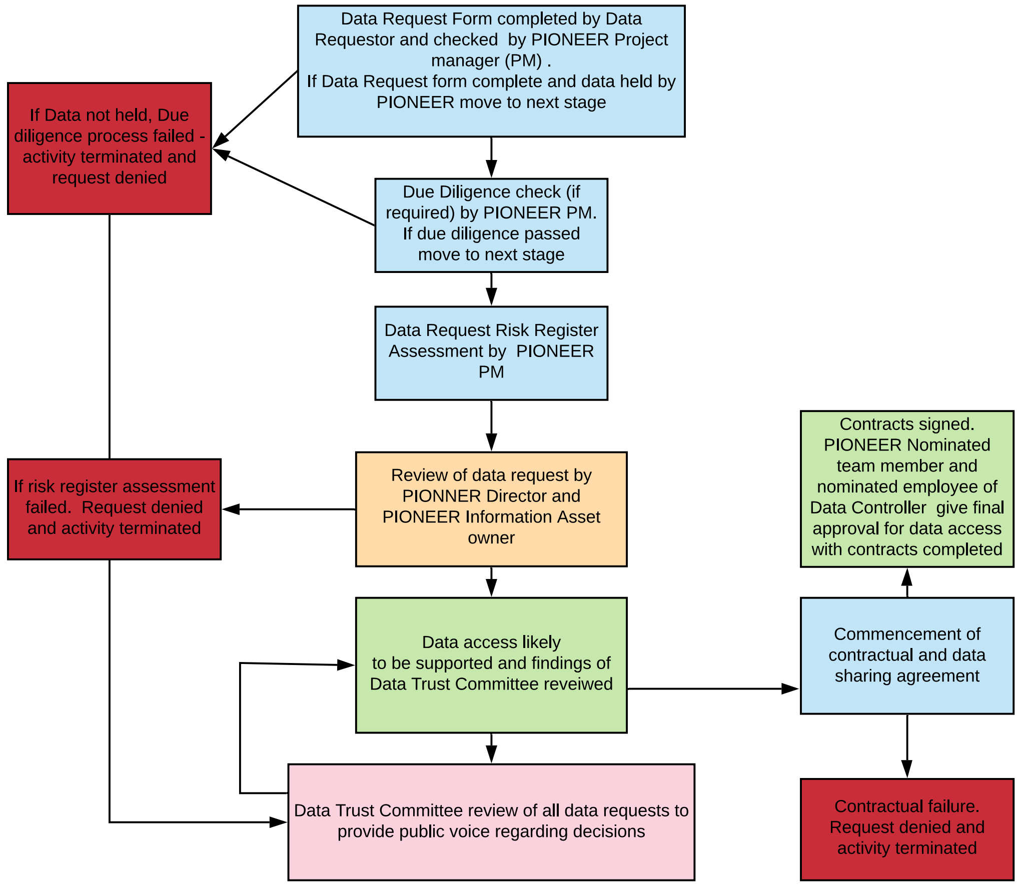


See the following sections for details of these processes.

| | |
|---|---|
| Name of form/ document | |



Gallier S, *et al. BMJ Health Care Inform* 2021; 28:e100294. doi: 10.1136/bmjhci-2020-100294

| | |
|---|---|
| Data Request Form | Box 1 and 2 |
| Due Diligence form and process | Appendix 2 |
| Data request risk register | Box 3 |
| Data Trust Committee processes | Section 6.2.1 and Figure 4 |
| Data Trust Committee Terms of Reference | Box 4 |

## 6.1 Access to Data Pathway

PIONEER's metadata catalogue and data dictionary will be freely available for researchers to browse to enable an understanding of the data held within PIONEER.

Data requests will be considered from organisations, companies, researchers, members of the public, or any agency or body; and for the purpose of the protocol, they are referred to as Data Requestors. All requests for access to data will be considered as part of a three-stage review and release mechanism.

These are:

- Stage one – Technical assessment – does PIONEER hold the data
- Stage two – Due Diligence, Data Request Risk evaluation and review- Are the "5 safes" met?
- Stage three – Contractual arrangements and data release.

While described in series, stage one and two may occur in parallel. Stage three cannot occur without the Director and PIONEER Data Controller (UHB approved and named delegates) approving. These processes are described in detail below.

All requests for licensed access to data will be considered against core principles for data access, and against the "5 safes" described in section 5.3:

1. Data requests that support a project which is likely to be of benefit to patients, to the NHS, or with clear societal benefits
2. Data requests are from organisations, researchers, individuals or companies which pass the Due Diligence process (see section 6.1.2.2)
3. Data requests which are ethical, appropriate, and include sufficient data to answer the proposed question but are not excessive in the data requested nor include data which has more than remote possibility of being re-identified by data held by the requestor or in the public domain. – i.e. Data requests which pass the risk evaluation.

Requests for access to data may be initiated through the HDR-UK Health Data Research Innovation Gateway or through direct contact with PIONEER team members. The process this initiates is the same for either means of contact.



Gallier S, *et al. BMJ Health Care Inform* 2021; 28:e100294. doi: 10.1136/bmjhci-2020-100294

The Gateway is an application which supports researchers and innovators to discover and access data from the UK Health Data Research Alliance in a safe and responsible manner, and contains a metadata catalogue of all data available through HDR-UK. Direct contact with PIONEER may be through its central website or email address. See Appendix 1.

All engagement will start with the Data Requestor completing a Data Request Form (DRF). The DRF also includes contact details and a description of what the request involves. The DRF should be sufficent to allow an assessment of the project, public good and "5 safes". Where more information is needed, this will be curated in a bespoke data request application, adding to the information within the DRF as needed.

**Box 1. PIONEER Data Request Form (indicative content)**

PIONEER Health Data Research Hub **Data Request Form**

**SECTION A: THE PROJECT**

**A1**: **Project title.** (200 characters)

**A2**: **Research question(s) and aim(s)** (up to 200 words)

**A3: Background and scientific rationale of the proposed research project** (up to 300 words)

**A4: A brief description of the method(s) to be used** (up to 300 words)

**A5: The type and size of dataset required** (up to 100 words)

**A6: The expected value of the research** (considering the public interest requirement) (up to 100 words)

**A7: Up to 6 keywords which best summarise your proposed research project**. (added here)

**A8: Lay Summary.**
A lay summary of your research project in plain English, stating the aims, scientific rationale, project duration, and public health impact suitable for publication on the PIONEER website (up to 400 words)

**A9: Have patient or public groups been involved in this project? If so, how? If not, why not?** (up to 400 words)

**A10: Will the research enhance the PIONEER Research Database by adding data fields or analyses?** Potential examples include derived analyses of existing data, new labels for data sets, or new analyses of data sets.



Gallier S, *et al. BMJ Health Care Inform* 2021; 28:e100294. doi: 10.1136/bmjhci-2020-100294

Yes/No

(if yes – add details – up to 300 words)

**A11: The estimated duration of the project, in months. (add here)**

**A12: How will results be shared / disseminated?** (up to 300 words)

**SECTION B: THE DATA, SETTING, AND ANALYSES**

**B1: Level of data access requirement**

a) Do you wish to commission PIONEER to conduct the analysis for you minimising your direct exposure to the data? Yes/No

b) Can you undertake the planned project using aggregate data only? Yes/No

c) Do you wish to request access to anonymised individual patient-level data? Yes/No

**B2: Selection of data-fields**

a) Standard data-fields requested (listed within the PIONEER Metadata catalogue). Yes/No
(List data fields required).

b) Additional data-fields requested (subject to availability). Yes/No
For additional data fields please identify their source (where known), for example are the fields:

i) collected as part of routine care within the NHS but are currently held outside of the existing NHS PIONEER partners?

ii) collected as part of linkage to external datasets (please specify which external datasets)?

**B3: Data environment:**

a) Will you access the data solely within the PIONEER Data Safe Haven? Yes/No

b) Will you require transfer of data to an alternative secure environment in order to achieve the project aims? Yes/No

c) If yes, then:
   i) What are the reasons that this transfer is required?
   ii) Are the standards of transfer ISO27000 series compliant?
   iii) Does the alternative Data Environment satisfy all requirements of:
      ISO27001 Yes/No
      NHS Data Security and Protection Toolkit Yes/No

**B4: Statistical analysis**

i) What forms of statistical analysis are planned? (up to 100 words)

ii) How is it intended that this will be presented in the final output? (up to 100 words)

iii) What is the smallest cell value that is likely to be generated by this analysis, and how will this be managed to avoid disclosure? (up to 100 words)



Gallier S, *et al. BMJ Health Care Inform* 2021; 28:e100294. doi: 10.1136/bmjhci-2020-100294

**B5: Machine learning**

a) Will the data be subject to any machine learning (ML) techniques? Yes/No

If Yes, please specify:

b) Type of ML technique(s)

c) Is the PIONEER data for:

i) Algorithm generation and training Yes/No

ii) Internal validation Yes/No

iii) External validation Yes/No

iv) Other - please specify

**B6: Ethical approvals**

a. Do you seek for your project to be approved under the generic favourable ethical opinion of the PIONEER Research Database (REF xxx)? Yes/No

b. Do you seek for your data access request to be considered under pre-existing ethical approval? (Please attach all relevant documents) Yes/No

**SECTION C: THE APPLICANT AND RESEARCH TEAM**

**C1: Lead Applicant**

i) Name
ii) Email address
iii) Current position
iv) Institution
v) Specific role(s) in the project

**C2: Evidence of Lead Applicant's expertise and experience relevant to delivering the project including:**

i) relevant publications (up to 5 most relevant)
ii) other relevant outputs

**C3: Sponsoring organisation**

i) Name
ii) Legal name (if different; to appear on any legal documents)
iii) Sector
iv) Size of institution

**C4: Co-applicants**

i) Name
ii) Current position
iii) their Institutions
iv) Specific role(s) in the project

**C5: Other significant project team members**

i) Name
ii) Current position
iii) their Institutions
iv) Specific role(s) in the project

**C6: Contact person**

i) Name



Gallier S, *et al. BMJ Health Care Inform* 2021; 28:e100294. doi: 10.1136/bmjhci-2020-100294

| |
|---|
| ii) Email address<br>iii) Preferred telephone contact number |
| **Internal Use only:**<br>**Log number:**<br>**Date request received:** **Time:**<br>**Due Diligence Log Number:** |

### 6.1.1 Stage One - Technical assessment

Each DRF request is logged by the PIONEER Project Officer with a unique number, and the date and time of the request. This information will be recorded on a Data Request Database, which will capture how the data requests flow through the PIONEER system, and the rationale and end results for final decision making. The DRF is also used to initiate due diligence checking and logged with a due diligence number, and to inform the risk assessment (Stage 2 of the process)

The DRF will be assessed against the following criteria (as described in Box 2)

## Box 2. DRF by PIONEER Operations Team

**Box 2 : Initial Screening of DRF by PIONEER Operations Team**

**PART A:**
SUFFICIENT INFORMATION SCREENING

A1) Is the form complete?
A2) Is the potential for patient benefit/public interest present and clearly stated?
A3) Is the data request clear (including number, types of data fields)?
A4) Is enough detail provided for reviewers to evaluate the extent to which the 'five safes' are met?

*If the answer is NO to any of the above questions, then register as an enquiry and return to the applicant for further information.*

*If the answer is YES to all questions, then register as a full application and proceed to part B.*

**PART B:**
PURPOSE SCREENING
B1) Is there potential for patient benefit/public interest?

APPLICANT DUE DILIGENCE SCREENING
B2) Does the applicant pass the PIONEER Due Diligence Process (Appendix 2)?

TECHNICAL SCREENING
B3) Is the data for which access is requested currently or potentially available within the scope of the PIONEER Research Database?
B4) Is access to the data requested legal?
B5) Is it feasible within the resources available to provide access to any/all of the data and services requested?



Gallier S, *et al. BMJ Health Care Inform* 2021; 28:e100294. doi: 10.1136/bmjhci-2020-100294

*If the answer is NO to any of the above, the application should be declined and the reasons given to the applicant.*
*If the answer is YES, proceed to Risk evaluation.*

#### *6.1.1.1 Data not within the PIONEER platform*

If the data does not exist within PIONEER, this will be fed back to the requester and the enquiry will be closed. The data request will be fed back to the PIONEER management team to determine if such data is within PIONEER's scope and should be considered for inclusion within PIONEER (for example, meteorological, air quality data, or pollen counts which are likely to impact upon acute care). Should the Management Team decide these data would enhance the PIONEER data offer, data discovery plans would be implemented to ascertain where such data assets exist and how these could be incorporated into the PIONEER data offer, either within PIONEER or through partnership working. All partnerships working with PIONEER would be expected to operate in accordance with the PIONEER protocol for the purpose of that partnership, and as stated in the relevant data sharing agreement.

### 6.1.2. Stage Two – Due Diligence

PIONEER will undertake due diligence checking for all Data Requestors, in recognition of the need for public trust in PIONEER operations. All companies will be checked to ensure they are not subject to UK financial sanctions; this information can be found at https://www.gov.uk/government/collections/financial-sanctions-regime-specific-consolidated-lists-and-releases. Individuals, organisations and companies who pass the due diligence checks will be provided with a Due Diligence Code. The due diligence check will be updated at each data request from that requestor. See Appendix 2 for the due diligence process but in brief:

The Due Diligence process consists of:

1) Checking the Due Diligence Code and assessing any previous due diligence checks.
2) Completing the necessary sections of the due diligence paperwork.
3) Researching predefined online media sources by keyword search.
4) Checking due diligence outcomes of HDR-UK gateway or other data providers
5) Generating the Due Diligence Code and circulating to key stakeholders.
6) Maintaining a log of all of the above.

PIONEER will follow the due diligence process as described in Appendix 2. All successful due diligence checks will be shared with HDR-UK. Any failed due diligence checks will result in a formal response to the Requestor; responses will not provide specific detail.



Gallier S, *et al. BMJ Health Care Inform* 2021; 28:e100294. doi: 10.1136/bmjhci-2020-100294

### 6.1.3. Stage Two – Further Information

Once the DRF is completed, it will be checked by PIONEER staff against criteria shown in Box 2. If the organisation has passed due diligence, more information may be required to understand the Data requestor's data needs or the Data Request Form may need amending. Version control of each Data request (DRF number, data and version number) will allow amended forms to be reviewed against previous application. Amendments may include PIONEER expert services, such as workshops with relevant patient groups, advice from healthcare practitioners, the curation of a bespoke dataset, algorithm generation, or an analytic plan. This will occur in discussion with a member of the PIONEER engagement team working with the Data Requestor. Needs analysis will be captured as part of a bespoke addition to the DRF form, and no specific template exists for this.

### 6.1.4. Stage Two – Risk Evaluation

Each DRF and outcome of due diligence will be reviewed by the PIONEER Operations Team and those forms which have passed these initial steps will be given a Data Request Risk Rating: green for low risk, amber for moderate and red for a failed risk assessment. The rating given will be based on the data requested, timelines, potential for reputational risk, and potential for patient gain, as outlined in Box 3. This will then be reviewed by the PIONEER Director, Information Asset Owner (or delegated staff member), and Data Trust Committee.

The following Risk Rating will be applied to all data requests and will include past dealings with PIONEER, PIONEER partners, or HDR-UK. Potential breaches of contract from past data use will be reviewed on a case by case basis and assessed for seriousness by the PIONEER Director, and the Data Controller's information governance legal and contracts department, as required.



Gallier S, *et al. BMJ Health Care Inform* 2021; 28:e100294. doi: 10.1136/bmjhci-2020-100294

**Box 3. Data Request Risk Rating**

| Descriptor | Green/ Low | Amber/ Moderate | Red/ High |
|---|---|---|---|
| Previous dealings? | Select one of:<br>**Yes.** Met all contractual obligations for data use, attribution, data security and outputs and acted in accordance with PIONEER guiding principles<br><br>Or:<br>**No** previous dealings but considered low risk of contractual breach<br><br>(add detail as needed) | Select one of:<br>**Yes;** previous dealings. Met contractual arrangements but minor deviations from PIONEER guiding principles (for example, open access)<br>Or: **No serious breach of contract** and no repeated breaches of contractual obligations<br>Or: **No previous dealings** but considered minor risk of contractual breach<br>(add detail as needed) | Select one of:<br>**Yes**: One or more serious breaches of contract or repeated breaches of contractual obligations<br><br>Or:<br>**No previous dealings** and considered high risk of contractual breach<br><br>Or:<br>**Previous serious contractual breach** with other HDR-UK data provider<br>(add detail as needed) |
| Data Use Summary | Clear potential for patient, NHS, or societal benefit<br>(add rationale) | Potential for patient, NHS, or societal benefit<br>(add rationale) | No potential for patient, NHS, or societal benefit<br>(add rationale) |
| Data Description | Data which is aggregated or highly unlikely to lead to patient identification<br><br>(add comment) | Data which may have a realistic potential for identification (for example, in the case of rare diseases or through the combination of data requested).<br><br>(add comment) | Data which has a realistic potential for identification because requestor holds an existing data set which may make identification possible.<br>(add comment) |
| Data security | Provides evidence of data security measures which meet all requirements<br><br>(add comment) | Provides evidence of data security measures which meet most requirements with additional support<br><br>(add comment) | No evidence of data security or evidence to suggest risk of data breach<br><br>(add comment) |
| Potential for reputational risk to PIONEER or HDR-UK | Low<br><br>(add rationale) | Moderate<br><br>(add rationale) | High<br><br>(add rationale) |
| Suggestion by PIONEER Team | Suggestion to support data release | Suggestion to support data release | Suggestion not to support data release |

These recommendations will be passed to the PIONEER Director and Information Asset Owner (or delegated staff members), and will be reviewed by the Data Trust Committee and PIONEER Management Team. The risk rating will inform the recommendation for data release, but will be a recommendation and not a binding outcome.



Gallier S, *et al. BMJ Health Care Inform* 2021; 28:e100294. doi: 10.1136/bmjhci-2020-100294

Where the suggestion is to support data release, contractual arrangements including data licence agreements and costs can be initiated, but not completed without PIONEER Director and Information Asset Owner approval.

## 6.2 Stage 2: Data Access Decisions and Data Trust Committee Processes

The PIONEER Director and Information Asset Owner (or delegated staff members) will review the risk rating, and document whether they will provisionally approve or definitively decline the access request, and record any further actions that may be needed. If a request has been declined due to a failed due diligence or risk evaluation, this will be fed back to the applicant and the request will be closed. Further applications will be accepted by the same data requestor only where the due diligence process had been passed or where there were substantial changes to the data requestor which meant a further due diligence review is warranted.

Provisional approval at this stage will not constitute full approval, which can only be given at stage 3, once Data Licence Agreements and Contracts are in place.

All requests for data access will be reviewed by the Data Trust Committee (DTC), including the DRF, Due diligence and risk evaluation outcomes and provisional opinion of the PIONEER Director and Information Asset Owner.

This information will be considered prospectively but then we aim over time to build criteria for proportionate review, which might allow retrospective DTC review for data release. See Figure 4 and section 6.2.1

### 6.2.1 Stage Two – Patient and Public Involvement in PIONEER Data Access Processes: The Data Trust Committee

The Data Trust Committee (DTC) is an advisory function for PIONEER and cannot approve data release (this can only be provided by the Information Asset Owner or their nominated delegate). The Data Trust Committee's Terms of Reference, make up, and meeting arrangements are described in section 6.2.1.1.

The DTC will review all data request forms, processes, decisions and outcomes.

Initially, for the first 12 months and until a substantial amendment is made to the protocol and ethics committee, it is envisioned that all data requests will be reviewed in detail prior to data



Gallier S, *et al. BMJ Health Care Inform* 2021; 28:e100294. doi: 10.1136/bmjhci-2020-100294

release. This will allow shared learning of processes, and the potential for benefit and risk of data release to be evaluated and discussed based on real world cases. However, as more data requests come into PIONEER, it is envisioned that there may be too many data requests for each to receive a full review by the DTC. A summary review may be sufficient for most low risk data requests, and this could occur either before data release or afterwards, to ensure the decision of the Director and Information Asset Owner is supported by the DTC. The criteria for this “proportionate review” must be developed by our DTC and have ethical approval, but this process is planned for the future.

The DTC will review in full the DRF, due diligence outcome, risk rating, and proposed actions of data requests. As stated in the terms of reference for the DTC (see section 6.2.1.1), the DTC will report a consensus decision of whether a requested data release should be supported or not, as shown in Figure 4. An executive summary of decision making and main points will be reported to the PIONEER team and Information Asset Owner or delegated staff member. The DTC will produce reports every 6 months to the PIONEER Strategic Executive Team (SET: see section 7.5). The DTC reports will be publicly accessible upon request and a lay summary of the report will be placed on the PIONEER website.

The DTC review will occur prospectively, prior to a decision for likely data release (and this is how the DTC will operate for the first year of PIONEER data access requests). In time, we envisage that the DTC can also review retrospectively, after data release has occurred, for releases which fall within the low risk category – but this can occur after the first year of PIONEER data access requests, with an agreed proportionate review criteria, and after appropriate ethical approvals.

Where prospective, the opinion of the DTC will inform the Data Controller and Information Asset Owner’s decision. A prospective decision by the DTC not to release data will prevent data release.

Retrospective assessments have been suggested following public and patient consultation for the following reasons;

1. There is likely to be a high number of data release requests, and these must be prioritised by potential risk to facilitate timely decisions.
2. Many data release requests will be considered low risk, and a prospective DTC review may be considered disproportionate to the risk of the data request.
3. Many data release requests will form a refreshed subscription dataset (where initial data release has been agreed, but the Data Requestor now wishes for a more up to date dataset)

This will allow the DTC to focus on data requests considered to be of medium risk. However, we will not perform retrospective reviews without specific ethical permission to do so and with a protocol amendment. The process below describes how this might happen.

All decisions of the DTC will be regarded as opportunities to learn and improve operational decision making within PIONEER, so that they reflect patient and public priorities and concerns, or to amend risk assessment criteria.

Where the DTC consensus for data release and Data Controller/ Information Asset Owner's actions are in agreement, this will be documented.

Where the DTC consensus supports data release but the Data Controller and Information Asset Owner do not support data release, this will be discussed to ensure there is learning around the decision pathway. The final decision however, remains with the PIONEER Director, Information Asset Owner or delegated staff member.

Any data release decisions where the DTC consensus decision was to decline data release but the Data Controller favoured data release will initiate a Data Trust Learning Review (DTLR). A DTLR must be convened within one month of the DTC decision. See section 6.2.2.

PIONEER operating procedures may be amended over time to reflect the learning gained from working closely with the DTC.

#### *6.2.1.1. DTC Terms of Reference*

The DTC will be established and terms of reference agreed to, as described below. In essence, the DTC will act as the public conscience of PIONEER and consider all data requests as described in section 6.2, Figure 3 and Figure 4.

The DTC will be made up of at most nine individuals, who will have a term on the DTC of up to four years, with the potential to reapply for a further term. There will be an open application by letter to become members of the DTC following open advertisement on the PIONEER website. Members of the PIONEER team and HDR-UK public advisory group will assist with DTC selection. All members of the DTC must declare all relevant conflicts of interest, including any relationship to Data Requestors, or any stocks or shares held in relevant industry stakeholders. The DTC will be assisted by experts in data research, information governance, and UK data law; though these experts will have an advisory capacity only and will not be voting members of the DTC. There will be a nominated professional secretariat. There will be a DTC Chair.



Gallier S, *et al. BMJ Health Care Inform* 2021; 28:e100294. doi: 10.1136/bmjhci-2020-100294

DTC members must sign up to the terms of reference of membership. These are given in Box 4.

## Box 4. Terms of reference for Data Trust Board

Terms of reference include:

- List of names of DTC members on PIONEER website.
- Have a named Chair and Deputy Chair
- Meet at least quarterly (but more frequently is expected) to discuss data requests and operations of PIONEER.
- All data requests will be regarded as confidential as only the lay summary will be published on the PIONEER website
- Review all Data Requests, Due Diligence, risk forms, and data provision decision by the PIONEER Director and Information Asset Owner or delegated staff members.
- Form a consensus decision on each data request (i.e. support or not supporting data provision).
- A consensus will be formed by individual voting, but the decision to support or not support data provision will only be reported as a consensus view, and not by number of votes.
- All voting will be confidential and not discussed outside of the DTC.
- A quorum of at least half of the DTC (rounded up) is required for the DTC to convene.
- Attendees at each meeting will be documented.
- All decisions are to be made in accordance with the protocol and principles of PIONEER as laid out in the protocol.
- The DTC will report their consensus decision and reasoning to the PIONEER team.
- The DTC will form a six-monthly report to the Strategic Executive team (SEG) and contribute to the annual REC review.
- The DTC will generate lay summaries of their activity for public review.
- The PIONEER Operations team and PPIE lead will assist in writing all reports for the DTC.
- All reports will be approved by the DTC prior to release to relevant groups.
- An exceptional DTC meeting can be called to consider urgent applications. Applications will only be considered urgent if they have significant and real time constraints which mean urgent data release is required. For example, at time of pandemics or outbreaks – where the acute care data set could help model responses, or if patient care appears compromised and data release could prevent harm to patient groups. The Director will suggest if an exceptional DTC meeting should be called, and the DTC Chair (or deputy) will decide if the DTC should be convened.



Gallier S, *et al. BMJ Health Care Inform* 2021; 28:e100294. doi: 10.1136/bmjhci-2020-100294

**Figure 4. Data Trust Committee Review Procedures**

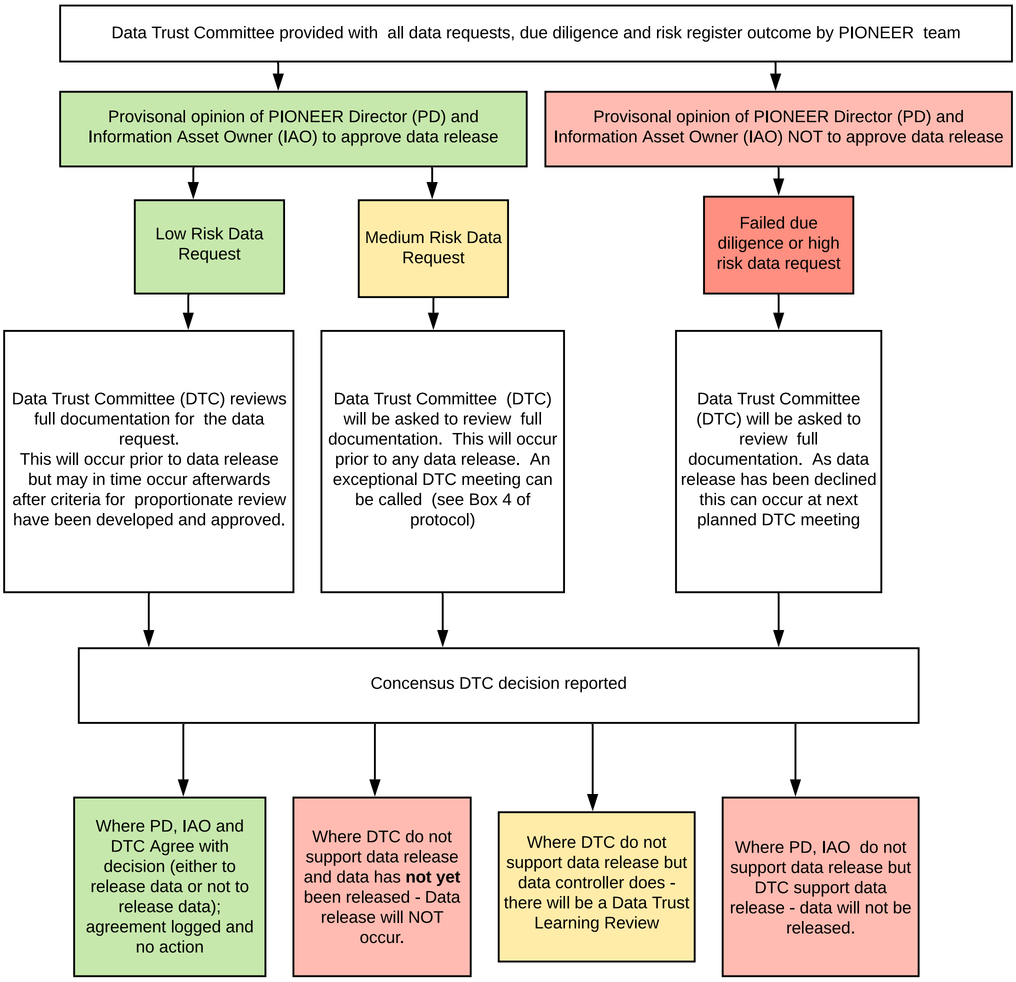


### 6.2.2. Data Trust Learning Review

Where the DTC consensus decision is that they would not have supported data release but the data Controller supported data release, a Data Trust Learning Review will be convened. This is a meeting which is chaired by the Strategic Executive Group Chair, and includes the Information Asset Owner, Director and Co-Directors of PIONEER, DTC Chair, DTC, and a representative of the Data Providers. A representative of the Ethics Committee who gave approval to the project and a representative of the HDR-UK Public Advisory Group will be invited but attendance is not compulsory. Here, the decision pathway for data release and DTC review will be discussed in general, including concerns, potential risks, and benefits for data release. This will be a learning experience and all aspects of decision making will be discussed with agreed action points. A report of the DTLR will be fed back to the Ethics Committee, Data Controller, and SEG including agreed action points for future operations.

## 6.3 Stage 3. Record and Release

No data release can occur without approval following the PIONEER ethics and governance processes.

Data release will require a Data Sharing Agreement (DSA), an associated and agreed costing model, and scheduling for follow up events (such as publication of data requests and actions, requests for data destruction, and audit).

Contractual arrangements (including those which are financial) must be approved by the Data Controller prior to data release. Data would then be released as agreed within the provisions of the DSA.

### 6.3.1 Specific Ethics Committee Approval of Research Projects

Where Sponsors approach the Research Database with pre-existing ethical approvals, the Sponsor will provide any/all necessary documentation to enable the technical and due diligence assessments. If the proposed project covers the data requested, then the Data Controller and Information Asset Owner (or approved delegate) will consider releasing the data in accordance with Stage 3 procedures.

### 6.3.2 Conditions of Data Release to Other Researchers

#### *6.3.2.1 Open Access*

Open access means that anyone with an internet connection can access the output of research, be it a journal article, algorithm, or methodology, without the need to pay for access via a subscription or other mechanism.

PIONEER operates with the following guiding beliefs about open access:

- Transparency is a PIONEER core value.
- PIONEER receives funding from the government and charitable organisations. It therefore acts for the public good, and must deliver value for money to the taxpayer and/or charitable donors.
- By being open, we can share more and learn quicker from each other's successes and failures. Open access makes research more transparent, rigorous and efficient; stimulates innovation; and promotes public engagement.
- The public voice is at the heart of all we do – non-researchers must be able to access the outputs of PIONEER research.

**PIONEER will operate within the following open access principles**



Gallier S, *et al. BMJ Health Care Inform* 2021; 28:e100294. doi: 10.1136/bmjhci-2020-100294

Noting the above, PIONEER:

- Expects authors to maximise the opportunities to make their results available for free and to encourage data outputs to be publicly accessible with lay summaries freely available.
- Expects outputs of work supported by PIONEER to select publishing routes that ensure the work is available immediately on publication in its final published form, where possible.
- Encourages authors and publishers to licence research papers using the Creative Commons Attribution licence (CC-BY), so they may be freely copied and re-used (for example, for text- and data-mining purposes or creating a translation), provided that such uses are fully attributed.
- Encourages outputs published in a peer-reviewed journal, and supported in whole or in part by PIONEER, to be made available through PubMed Central (PMC) and Europe PMC as soon as possible, and in any event within six months of the journal publisher's official date of final publication.

#### *6.3.2.2 "Public Good" Condition for data release*

All requests for data must have demonstrable potential for public benefit. This includes but is not limited to;

- The development of new health care processes, pathways, biomarkers, devices, therapeutics, and software as medical devices.
- The development of new NHS services or new models of health care, and development of new or augmented social care.
- Benefit to the NHS, through products, services, regulatory reports, audits, or direct and indirect financial benefits
- Benefit to the public through the creation of new knowledge, products, or services.

The Data Trust Committee will review all data provision decisions against the condition of public good, as described in section 6.2.

#### *6.3.2.3 Attribution Policy*

PIONEER research outputs include typical academic measures of success, such as publications. As publications are increasingly announced on social media platforms, such as Twitter and LinkedIn, attribution of tweets is also set out in this protocol. Core to HDR-UK's mission and PIONEER policy is the generation of algorithms, code, software, and methodologies that facilitate the analysis of large-scale data, so these are also covered by this protocol.



Gallier S, *et al. BMJ Health Care Inform* 2021; 28:e100294. doi: 10.1136/bmjhci-2020-100294

For publications and communications, PIONEER must be included in acknowledgements or the funding section. Suggested text includes:

- *This work was supported by PIONEER, the Health Data Research Hub in Acute Care, which is funded by Health Data Research UK.*

For code and related digital artefacts:

- PIONEER would encourage code (e.g. algorithms, analytical script, source code) and related digital artefacts (e.g. documents) to be made available within the HDR UK GitHub repositories.
- Otherwise, similarly liberal and open source licenses (such as Apache 2.0, BSD, Eclipse Public License) should be used, permitting anyone to benefit from, improve upon, and redistribute the code.

#### *6.3.2.4 Downstream security*

Data from the PIONEER research database will be released on condition that data will be held securely to the standards described in section 5.0 of this protocol, and its integrity will be maintained. The Data controller and Information Asset Owner may request evidence of systems, policies, or procedures to ensure such and this will be reflected in data sharing agreements.

### 6.3.3. Data Access Request Denied

The final decision for data access resides with the Data Controller and Information Asset Owner. All decisions will be clearly documented within the Data Request Database and a report generated. The general themes for data access denial will include but not be limited to:

1. Data is not within the PIONEER data set
2. Individual / organisation / company fails due diligence
3. Data request fails the public good condition of data release (see section 6.3.2.2)
4. Concerns about the data security, secondary uses, or risk of public harm – failure of the "5 safes"
5. Failure to form a data sharing agreement, or contractual failure

All reasons will be documented and the overall decision fed back to the Data Requestor. There are no procedures to challenge this decision, which is final. The Data Requestors can submit further data requests as desired. All decisions will be discussed with the Data Trust Committee and Strategic Executive Group, but the decision remains that of the PIONEER Director and the Information Asset Owner.



Gallier S, *et al. BMJ Health Care Inform* 2021; 28:e100294. doi: 10.1136/bmjhci-2020-100294

# 7.0 Ethical approvals, Management and Governance

## 7.1 PIONEER Management Committee (PMC)

PIONEER will convene a Management Committee (PMC). The PMC will meet at least quarterly and will be made up of the Director or Co-Director of PIONEER, Workstream Leads, a representative of the Data Providers, Industry Leads, a DTC Patient and Public Advisory Group representative, Business Engagement Leads, the Information Governance and Ethics Officer, the PIONEER Programme Manager, Contracts and Finance representatives, and a Secretariat. The terms of reference for the PMC are given in Box 5.

**Box 5. Terms of reference for the PIONEER Management Committee**

The PMC will:

- Keep an overview of the data within the database's control – version controlled
- Provide an overview and be able to demonstrate the monitoring of data security
- Develop an initial standard operating procedure to ensure compliance with the protocol
- Identify and document substantial amendments to the design, management or conduct of the research database for submission to the reviewing REC.
- Review potential data providers and make a recommendation of their addition to PIONEER to the Strategic Executive Group
- Review data requests and data requests outcomes and timelines
- Review outcomes from DTC
- Review contractual procedures and timelines
- Monitor and review cost recovery strategy and overall finances
- Monitor and review reports and ensure timely submission
- Commission, approve, and submit reports to the Data Controller and their delegated operator.
- Commission, approve and submit reports to the Strategic Executive Group
- Commission, approve and submit reports to the Ethics Committee and HRA
- Maintain a risk register

## 7.2 Strategic Executive Group (SEG)

PIONEER will convene a Strategic Executive Group (SEG). The SEG will meet at least six-monthly, and will be made up of the Executive Chair of PIONEER, Chief Executive Officer or Deputy for each Data Provider, representatives of Industry and academic partners, and the PPIE Lead. The Director or Co-Director of PIONEER and PPIE lead will report to the SEG, assisted by relevant members of the PMC. There will be a nominated secretariat.



Gallier S, *et al. BMJ Health Care Inform* 2021; 28:e100294. doi: 10.1136/bmjhci-2020-100294

The SEG will agree on their terms of reference, but these will include providing strategic oversight of potential data providers, policy, and procedural links to external agencies relevant to PIONEER, supporting PIONEER operations within partner organisations, agreeing any changes to PIONEER operational procedures, and overseeing DTLR meetings.

### 7.3. Ethical Conduct

We will be seeking ethical approval from the NHS Research Ethics Service (now a function of the Health Research Authority).

Consent is not the legal basis for using these data for research purposes. PIONEER will process data without the consent of patients, and is reliant on Section 251 approval provided by the Confidentiality Advisory Group. The purpose of this approval is to set aside the common law for confidentiality in processing this data, to render it pseudonymised and then anonymous for the purposes of providing data sets for research.

PIONEER will continue to work with the relevant teams on the emerging SOPs / processes to ensure compliance with the NHS Digital “Opt-out” policy and the relevant legislation around this. A privacy notice for this project will be developed and linked to the Trust main privacy notice, working with the Head of Research Governance. Whilst this is not required, this provides an enhanced degree of transparency.

The tasks performed will be in line with the research protocol. The data is not currently available or collected at scale, and this is the only reasonable way of collating the information; to support the advancement of patient care.

### 7.4. Research Governance

PIONEER will ensure that researchers are responsible for ensuring that research will be conducted according to this protocol and related written instructions, and that the research adheres to current applicable legislation. Agreements with the Trust at each participating centre will be in place covering data collection.

### 7.5 Reporting Breach of PIONEER Policy

Protocol non-compliance will be reported without delay to the Data Trust Committee, SEG, and Data Provider Partners. The UHB Data Controller will designate an individual who will ensure that the issue is investigated and appropriate actions are taken. The reviewing REC will be notified as soon as possible of any serious breach of the REC approval conditions, or any serious breach of

security or confidentiality, or any other incident that could undermine public confidence in the ethical management of the data. Information Governance data breach policies will be followed in accordance with UK law.

### 7.6. Progress Reports and Accountability

PIONEER and collaborating researchers share responsibility for providing accurate periodic progress reports, as required by the main REC, host NHS Trust, and other authorised agencies (such as funding bodies).

PIONEER will maintain a record of all research projects for which data has been released. The record should contain at least the full title of the project, a brief lay summary of its purpose, the name of the lead researcher, the approving body, the date of approval, and the approval reference number, together with details of the data released to the project. The main REC and host NHS Trust may request access to this record at any time.

An annual report will be provided to the main REC and NHS Trusts, listing at minimum the details of data collection activity, the details of all approved projects for which data has been released in the previous year, and any related publications. For the purpose of annual reports, PIONEER will standardise on a single anniversary date i.e. the date of favourable ethical opinion.

### 7.7 Funding and Infrastructure Support

PIONEER is funded by Health Data Research UK and the MRC by the UK Industrial Strategy Challenge Fund for the first 2 years. This includes funding for design and data management. PIONEER has secured supporting contributions, or funding in kind, for more than 50% of the funding requested over the lifetime of the award, and will seek further funding with due diligence to ensure it has the capacity for sustainability and growth.

This initial funding requires that PIONEER is self-sustaining by the end of the period, so as to enable it to continue to provide benefit beyond its initial investment.

HDRUK notes that “all hubs need sustainable business models that enable them to continuously improve the range and quality of data available to researchers for fair and ethical use, thus maximising the benefit to the public from this critical resource.” HDRUK and the seven hubs are working with patients, the public and potential users through a series of workshops and consultation events, as part of a national conversation to define what sustainability model(s) would be considered appropriate and deliver fair value to the NHS and its patients.
PIONEER is committed to transparency with regards to all funding arrangements.



Gallier S, *et al. BMJ Health Care Inform* 2021; 28:e100294. doi: 10.1136/bmjhci-2020-100294

The rationale for having a clear commercial framework for the Hubs is fourfold:

1. Promote public trust through transparency of commercial arrangements
2. Improve the data access user experience through a consistent and transparent model for users
3. Ensure that commercial arrangements serve the public interest
4. Provide a common language and enable Hubs and other organisations to collaborate and learn from each other as they develop sustainable business models. This may extend to the use of model contracts, terms, and terminology, drawing on lessons from the Health Research Authority (HRA) Commercial Model Clinical Trial Agreements, and lessons from other jurisdictions to share best practices and reduce time to develop agreements.

PIONEER will develop a funding model for data access requests based on the time taken to curate the dataset, the extra services that may be needed (patient or healthcare workshops, analysis, machine learning approaches etc.) and the requestor (NHS, academic, commercial – and if commercial Small/Medium enterprise or large enterprise).

## 8.0 Communication and Dissemination Policy

PIONEER is committed to open and transparent communications which will support and acknowledge patient and public input, help maximise access for high-quality collaborative research, and publicise research outputs.

### 8.1 Communicating and Promoting the Work of PIONEER

Patient and Public Involvement and Engagement (PPIE) is central to the design and delivery of PIONEER, and this - and its cross-sector representation of stake-holders - is reflected in its approach to communication and dissemination.

The existence of PIONEER will continue to be communicated through national and international health data research networks. Details about PIONEER are available via the internet using websites maintained by HDR UK, and it will also be publicised widely in regular reports to funding bodies and sponsors. PIONEER will engage the public and patient communities wherever possible, and will work with existing Industry and Trust structures to communicate and publicise its work.

### 8.2 Communicating and disseminating research output arising from PIONEER

PIONEER is committed to maximising the value of PIONEER to patients and the public through the publication and dissemination of research findings, whether positive or negative, from all studies



Gallier S, *et al. BMJ Health Care Inform* 2021; 28:e100294. doi: 10.1136/bmjhci-2020-100294

conducted on data from the PIONEER Research Database. PIONEER is committed to an Open Science approach and open access publication.

Researchers utilising PIONEER do so on the understanding that they intend to publish the research findings in specialist peer reviewed scientific journals. Results may also be presented at scientific meetings and used for a thesis or other legitimate purpose.

PIONEER recognises that the publication of some results may be delayed for commercial reasons; however PIONEER expects a commitment from all users including industry to publish all results (positive and negative) within an appropriate time frame.

Authors should acknowledge the support of PIONEER as appropriate and provide a copy of all publications to the PIONEER Leadership Team.

Standard text for inclusion in all publications arising from PIONEER will be provided by PIONEER. This specifically acknowledges the work of PIONEER and the contribution of the partner NHS trusts and their patients.

## 9.0 Ongoing PPI/E strategy for PIONEER

Public and Patient Engagement and Involvement (PPI/E) are central to all PIONEER operations. The PPI/E strategy has been developed with the PIONEER PPI/E group, and continuing outputs from the group will be co-created and made publicly available on the HDR-UK PIONEER website.

### 9.1 PPI/E Overarching Aims

1. Patients and the public are partners in PIONEER.
2. The needs, values and interests of patients and the public are understood and embedded in PIONEER executive decision making.
3. People have trust and confidence in the use of health data within PIONEER for research and innovation
4. People have tangible gains from their data being used in research and innovation as part of PIONEER

Please see Appendix 3 for the PIONEER PPI/E Strategy

## 10.0 Protocol Amendments

Any change in the PIONEER protocol will require an amendment which will require ethical review and approvals. Any proposed protocol amendment will be initiated by the PIONEER Director and agreed by the Information Asset Owner. Any required amendment documents will be circulated to the PIONEER Management group, PPI/E group, and SEG. The information asset owner and Director will sign any amended versions of the protocol.

## 11.0 Annual Reports and Dissemination of Findings

PIONEER and collaborating researchers share responsibility for providing accurate periodic progress reports as required by the main REC, host NHS Trust, and other authorised agencies (such as funding bodies).

PIONEER will maintain a record of all research projects for which data has been released. The record should contain at least the full title of the project, a brief lay summary of its purpose, the name of the lead researcher, the approving body, the date of approval, and the approval reference number together with details of the data released to the project. The main REC and host NHS Trust may request access to this record at any time.

Any publications arising directly from the PIONEER database will be reviewed, approved and written with the acknowledgment of PIONEER and HDR-UK support, with authorship following recognised international guidelines as described in the International Committee of Medical Journal Editors (17). Publications resulting from access to data (which will have been approved by the Data Access Committee, as described elsewhere) will be requested to acknowledge PIONEER as the source of such data, and where appropriate and by mutual agreement, to involve members of the PIONEER consortium as contributors to the design, analysis, or other input to the resulting work.

## 12.0 References

1. England NHS. Urgent and Emergancy Care. https://wwwenglandnhsuk/five-year-forward-view/next-steps-on-the-nhs-five-year-forward-view/urgent-and-emergency-care/ 2018; DOA 20.1.2020.
2. England. N. Delayed transfer of care data 2018 - 2019. https://wwwenglandnhsuk/statistics/statistical-work-areas/delayed-transfers-of-



Gallier S, et al. BMJ Health Care Inform 2021; 28:e100294. doi: 10.1136/bmjhci-2020-100294

care/statistical-work-areas-delayed-transfers-of-care-delayed-transfers-of-care-data-2018-19/ 2018; DOA 12 Feb 2019.

3. Wachelder JJH, van Drunen I, Stassen PM, Brouns SHA, Lambooij SLE, Aarts MJ, Haak HR. Association of socioeconomic status with outcomes in older adult community-dwelling patients after visiting the emergency department: a retrospective cohort study. *BMJ Open* 2017; 7: e019318-e019318.
4. Ladha KS, Young JH, Ng DK, Efron DT, Haider AH. Factors affecting the likelihood of presentation to the emergency department of trauma patients after discharge. *Ann Emerg Med* 2011; 58: 431-437.
5. Kannan VC, Rasamimanana GN, Novack V, Hassan L, Reynolds TA. The impact of socioeconomic status on emergency department outcome in a low-income country setting: A registry-based analysis. *PLOS ONE* 2019; 14: e0223045.
6. Hsia RY, Sabbagh SH, Guo J, Nuckton TJ, Niedzwiecki MJ. Trends in the utilisation of emergency departments in California, 2005–2015: a retrospective analysis. *BMJ Open* 2018; 8: e021392.
7. Chalmers NI. Racial Disparities in Emergency Department Utilization for Dental/Oral Health-Related Conditions in Maryland. *Front Public Health* 2017; 5: 164-164.
8. Pham TM, Gomez-Cano M, Salika T, Jardel D, Abel GA, Lyratzopoulos G. Diagnostic route is associated with care satisfaction independently of tumour stage: Evidence from linked English Cancer Patient Experience Survey and cancer registration data. *Cancer Epidemiology* 2019; 61: 70-78.
9. McPhail S, Elliss-Brookes L, Shelton J, Ives A, Greenslade M, Vernon S, Morris EJA, Richards M. Emergency presentation of cancer and short-term mortality. *Br J Cancer* 2013; 109: 2027-2034.
10. Herbert A, Abel GA, Winters S, McPhail S, Elliss-Brookes L, Lyratzopoulos G. Cancer diagnoses after emergency GP referral or A&E attendance in England: determinants and time trends in Routes to Diagnosis data, 2006–2015. *British Journal of General Practice* 2019; 69: e724.
11. Schurig AM, Böhme M, Just KS, Scholl C, Dormann H, Plank-Kiegele B, Seufferlein T, Gräff I, Schwab M, Stingl JC. Adverse Drug Reactions (ADR) and Emergencies. *Dtsch Arztebl Int* 2018; 115: 251-258.
12. Janssens K, McDonnell T, McCarthy G. Chronic Disease in the Emergency Department. *International Journal of Integrated Care* 2017; 17: A396.
13. National Institute for Health and Care Excellence. Emergency and acute medical care in over 16s: service delivery and organisation. https://wwwniceorguk/guidance/ng94 2018; DOA 20.1.2020.
14. Tomašev N, Glorot X, Rae JW, Zielinski M, Askham H, Saraiva A, Mottram A, Meyer C, Ravuri S, Protsyuk I, Connell A, Hughes CO, Karthikesalingam A, Cornebise J, Montgomery H, Rees G, Laing C, Baker CR, Peterson K, Reeves R, Hassabis D, King D, Suleyman M, Back T, Nielson C, Ledsam JR, Mohamed S. A clinically applicable approach to continuous prediction of future acute kidney injury. *Nature* 2019; 572: 116-119.
15. Desai T, Ritchie F, Welpton R. Five Safes: designing data access for research. https://www2uweacuk/faculties/BBS/Documents/1601pdf 2016; DOA 2.3.2020.
16. UKRI.org. Concordat on Open Research Data. https://wwwukriorg/files/legacy/documents/concordatonopenresearchdata-pdf/ 2016; DOA 7th Match 2020.
17. ICMJE. Defining the role of authors and contributors. http://wwwicmjeorg/recommendations/browse/roles-and-responsibilities/defining-the-role-of-authors-and-contributorshtml 2018; DOA 20.1.2020.

## Appendix 1. Innovation Gateway

The following is a description of the HDRUK Innovation Gateway as provided by HDRUK on their public webpages, https://www.hdruk.ac.uk/infrastructure/gateway/ .

Health Data Research Innovation Gateway

Gateway

The Health Data Research Innovation Gateway is an application which will support researchers and innovators to discover and access data from the UK Health Data Research Alliance in a safe and responsible manner.

Overview

The Health Data Research Innovation Gateway will act as a common portal through which researchers and innovators in academia, industry and the NHS can search for and request access to UK health research data held by members of the Alliance and the Hubs in Trusted Research Environments to provide a safe location for data storage and access. The Gateway will support the use of data, facilitate interoperability, and provide analytical capability. It will take the form of a common web application providing the following functions:

- The ability to search for available data
- The facilitation of access requests to multiple data custodians
- Integration with accredited Trusted Research Environments to provide secure access to linked datasets
- A library of curated analytics tools and scripts
- A dashboard to show usage and quality of datasets for research and innovation to provide transparency to data users, data custodians and the public

The Gateway will not store or hold health data. Data security is paramount and data will continue to be held by data custodians in Trusted Research Environments
The Gateway will be designed to operate at a national and international scale, and to be scalable as the uses of health data increases. HDR UK works in partnership with NHSX and other NHS bodies to ensure that the Gateway aligns with related NHS endeavours, including the development of clear standards for the use of technology in the NHS.

## Appendix 2. Due Diligence Form and Process

PIONEER Health Data Research Hub

# DUE DILIGENCE FORM

| | | |
|---|---|---|
| **Due Diligence Log Number (xxxx)** | | |
| **Date of Due Diligence Review:** | | |
| **PROPOSED FUNDER/PARTNER INFORMATION – Initial assessment** | | |
| 1a | Principal address of business funder/partner: | |
| 1b | Number of years (months if less than 1 year) the entity has been in existence? | |
| 1c | Any relevant parent/subsidiary companies and other affiliations? | |
| 2 | Is the entity involved in any aspect of the tobacco industry (including investment in/by the business)? | Yes/No<br>If YES please elucidate: |
| 3a | Is the entity involved in any aspect of arms manufacturing or trade? | Yes/No<br>If YES please elucidate: |
| 3b | Are you aware of any links between the entity and state governments, companies or individuals with current or past history of serious human rights violations? | Yes/No<br>If YES please elucidate: |
| 4 | Are you aware of any reputational or relational difficulties for PIONEER in entering in to the proposed relationship? i.e. damaging media interest? | Yes/No<br>If YES please elucidate: |
| 5a | Principal address of business funder/partner: | |
| 5b | Number of years (months if less than 1 year) the entity has been in existence? | |
| 5c | Any relevant parent/subsidiary companies and other affiliations? | |
| 6 | Within the last five years, is there any published evidence that the entity, any predecessor of the entity, or any member of the entity has been associated with any of the keywords listed in the 'check for controversies'? | Yes/No<br>If YES please elucidate: |
| 7 | **Any previous relationships with HDR-UK or PIONEER partners?**<br>If Yes –Were concerns highlighted? | **Yes/ No**<br>**If YES please elucidate** |

| | | Yes/No | Review Date |
|---|---|---|---|
| 8 | **Due Diligence outcome by PIONEER team member**<br>Yes – Data Requestor does not contravene any of the above statements<br>No – Due Diligence check failed is to be declined | | |

| **APPROVED DUE DILIGENCE CODE** | |
|---|---|

| **SIGNATURE** - Leading member of PIONEER Staff**:** | |
|---|---|
| Print Name: | |
| Date: | |

*Please note: The content of this form, and any attached due diligence documentation is subject to the Freedom of Information Act and the Data Protection Act. Please do not include any content that is unsuitable for dissemination*

**Special Form 1: Laws and agreements to consider in regards to arms manufacturing and trade**

| **Type of Arms** | **3ai. Does the entity manufacture or trade in this type of arms?** | **Relevant Treaty** | **3aii. If yes to manufacture or trade, does the entity comply with this treaty?** |
|---|---|---|---|
| Explosive projectiles weighing less than 400 grams | | Declaration of Saint Petersburg (1868) | |
| Bullets that expand or flatten in the human body | | Hague Declaration (1899) | |
| Poison and poisoned weapons | | Hague Regulations (1907) | |
| Chemical weapons | | Geneva Protocol (1925) | |
| | | Convention on the prohibition of chemical weapons (1993) | |
| Biological weapons | | Geneva Protocol (1925) | |
| | | Convention on the prohibition of biological weapons (1972) | |
| Weapons that injure by fragments which, in the human body, escape detection by X-rays | | Protocol I (1980) to the Convention on Certain Conventional Weapons | |
| Incendiary weapons | | Protocol III (1980) to the Convention on Certain Conventional Weapons | |
| Blinding laser weapons | | Protocol IV (1995) to the Convention on Certain Conventional Weapons | |



Gallier S, *et al. BMJ Health Care Inform* 2021; 28:e100294. doi: 10.1136/bmjhci-2020-100294

| | | | |
|---|---|---|---|
| Mines, booby traps and "other devices" | | Protocol II, as amended (1996), to the Convention on Certain Conventional Weapons | |
| Anti-personnel mines | | Convention on the Prohibition of Anti-Personnel Mines (Ottawa Treaty) (1997) | |
| Explosive Remnants of War | | Protocol V (2003) to the Convention on Certain Conventional Weapons | |
| Cluster Munitions | | Convention on Cluster Munitions (2008) | |

## 1. Researching online media sources to identify controversies

The most significant aspect of the due diligence process will be to undertake research online to screen for controversies.

If any relevant parent/subsidiary companies have been identified, these must also be researched.

➢ ***How do I carry out an online search to check for controversies?***

The below keywords/phrases should be used to carry out Google searches on prospective corporate funders against a pre-defined list of sources. Where these identify potential controversies, ad-hoc searches may also be used to research these further.

| Keyword/phrase |
|---|
| Ethical |
| Abuse |
| Bribery |
| Controversy |
| Corporate Manslaughter |
| Corruption |
| Discrimination |
| Extremism |
| Financial Irregularity |
| Fraud |

| Keyword/phrase |
|---|
| Human Rights |
| Illegal |
| Litigation |
| Slavery |
| Tobacco |
| Arms Trade |
| Defence |
| Trade Embargoes |
| UN sanctions |
| Health and Safety Breach |

Proscribed list of credible sources (October 2018):

- www.reuters.com
- www.bbc.co.uk
- www.wsj.com
- www.economist.com
- www.nytimes.com
- www.theguardian.com

For example, for a funder called *Paradigm Shifting Research Funding Ltd*, the Google search terms used would be:

- "Paradigm Shifting Research Funding Ltd" bribery
- "Paradigm Shifting Research Funding Ltd" controversy
- "Paradigm Shifting Research Funding Ltd" "corporate manslaughter"
- …etc.

***Tip**: Use the 'Revised DD Google tool' spreadsheet saved HERE to generate the full list of search terms. Once generated copy & paste the list into the 'Multiple Tabs Search' Chrome browser extension. (Add this extension to your browser using the following LINK)*

➢ ***What counts as a controversy?***

The keywords act as a helpful guide as to what constitutes a controversial issue. Any media coverage relating to any of these issues has the potential to negatively impact on PIONEER if funding is accepted, and as such should be recorded as part of the due diligence review.

➢ ***Why is tobacco included as a keyword?***

The University's Code of Ethics states "The University's investment policy excludes direct investment by/in the tobacco industry." This is due to the terms of our agreement with Cancer Research UK (CRUK). Any investment in/from a company involved in the production of tobacco or tobacco-related products (i.e. cigarettes, etc.) could jeopardise our contract with CRUK. Thus it is imperative that any link between a company offering funding and tobacco is included in the due diligence paperwork, however small. This can be as apparent as a cigarette manufacturer, or as subtle as a company supplying machinery to that manufacturer.

➢ ***How should findings be recorded?***

The role of the researcher is to present an objective, rounded summary of any news stories which point to controversies. This may require including some contextual background to findings so that, when it comes to sign-off, findings are conveyed fairly and accurately.

As an example, imagine a large pharmaceutical organisation is offering funding to PIONEER for data access. In carrying out research, you discover that the company is in ongoing litigation. This should be included in the findings, but the specific nature of the lawsuit will also have a bearing on the decision to accept or reject funding. If the lawsuit is in relation to claims over the side effects of Powercetemol in earlier trials, this will have a material bearing on the due diligence. On the other hand, if the lawsuit is in relation to a different drug altogether, or a shareholder dispute, or anything unrelated to the proposed PIONEER research project, the impact is less acute. Context, therefore, is clearly very important when recording findings, and will prove helpful when it comes to making a final risk assessment at the point of sign-off.

➢ ***What timeframe should be considered when researching findings?***

Generally speaking, any reports in the past five years should be considered when researching controversies. However, if a matter of particular concern is identified outside of this timeframe, it should be included.

➢ ***Do I need to include references?***

Yes, footnotes linking to the news articles discovered should be included. Remember, even reliable sources need to be treated with care, so it is best practice to 'dual source' wherever possible. This is the act of locating a second article from a separate reputable publication which covers the same issue, and adds to the rigour of findings by presenting multiple touch points.

## Appendix 3 PPI/E Strategy

**Strategy Development Overview**

**Defining our principles**

Based on our engagement and involvement to date, we outline nine principles as a basis for further reflection and development to underpin our strategy:

1. Patients and the public are involved in making decisions about how health data is used in PIONEER and will continue to be involved throughout the programme
2. The benefits to patients and the public will be explicitly demonstrated in all research and outputs coming out of PIONEER
3. The involvement of patients and the public is acknowledged in all project summaries provided by PIONEER and in all research outputs from PIONEER
4. Information about the requests for research data access and the proposed reasons for use will be published by PIONEER
5. All requests for data will include a description of public and patient involvement in the research and will form part of the evaluation criteria
6. All PIONEER data users will provide accessible summaries of research
7. We will ensure PPI/E activity is inclusive and reflects the diversity of the UK
8. We work to increase awareness and understanding of how health data can be used in research and discuss data use transparently to increase trust
9. We will be open and transparent when things go wrong. We will learn from these experiences to mitigate future risk and explain what we have learnt, openly.

**Defining our stakeholders**

We want to include a diverse range of interests, experiences and voices in our strategy and PIONEER's delivery, noting that:

- PIONEER will include data from patients with chronic illnesses who may be very familiar with NHS healthcare, research and health data use
- PIONEER will include health data from people who may have experienced a sudden event (such as an infection) with less experience of healthcare
- PIONEER will include health data from a range of older adults (who make up a major and increasing proportion of acute care) who may have little experience of the concepts of health "data"
- PIONEER will include data from children and those aged over 13 may opt out of data sharing
- PIONEER will include data from people from different cultures and backgrounds
- Our PPI/E work needs to reflect this and be inclusive and accessible to all

**Defining our existing assets and partners**

We have a number of active initiatives ongoing across the involvement and engagement agendas which we can utilize to enhance and accelerate PIONEER's work:

- Patient steering groups already contribute to PPI activities across NHS data providers and there is now a cross-UHB/UoB PPI Steering Group which includes both children (Young Persons' Advisory Group) and adults, supported by specific training to increase PPI capacity and capability.
- Birmingham's NIHR-funded Clinical Research Facility (CRF) has an existing programme of Research Ambassadors who interact with local groups to increase awareness of research in under-represented communities, and have specifically helped to enhance participation from BAME groups.
- To provide complete transparency and understand better what people want from their health data, UHB is about to start a 'Universal Consent' research study asking people how they would like their routinely collected health data used, and flagging their preference on their Electronic Health record.
- Birmingham also hosts the INSIGHT Health Data Research Hub, and we have agreed to jointly coordinate involvement and engagement approaches to add value to each other's work. We also plan to consult HDR UK's own Public Advisory Board for a coordinated perspective on the wider health data landscape



Gallier S, *et al. BMJ Health Care Inform* 2021; 28:e100294. doi: 10.1136/bmjhci-2020-100294

**Defining ongoing involvement in PIONEER's structure**

Long-term, valuable and valued representation and a clear voice for patients and members of the public across PIONEER's committees will be a vital characteristic:

- Mr Gary Price – our nominated PPI/E co-applicant and lead – will chair a Data Trust Committee with support from PIONEER's PPI/E Manager.
- The DTC will consist of patients and members of the public who will apply to join, and then agree a Terms of Reference including tenure of members. The DTC will review and contribute to all executive decision making.
- DTC will have sitting members on the Data Governance Committee, Management Committee and DTC will receive the minutes from these meetings. The DTC will discuss progress, data applications and Mr Price feedback the DTC discussions to the Executive Committee, of which he would be a member.
- DTC will co-create and co-deliver public events about PIONEER, review the process/progress of data curation across partners (with appropriate support) to ensure we meet ICO principles of lawfulness, fairness and transparency in data curation.
- In line with the cross-UKRI Public Engagement strategy, PIONEER will have a major focus on enabling businesses to engage more effectively with patients and the public. DTC will work with academics and clinicians to set up an Expert Service offer training businesses how to engage with patients in the design, delivery and dissemination of innovation.
- PPI/E time/costs will be reimbursed in accordance to INVOLVE principles.

**Defining our approach to informing and refining our long-term PPI/E strategy**

Alongside the recruitment of our DTC, we will consult with a wide diversity of other stakeholders to inform our core PPI/E strategy, utilizing a range of mechanisms of engagement:

- We will increase visibility of the opportunities and challenges for health data use for patients, the public and NHS staff by holding a series of public facing events within NHS data provider facilities.
- We will hold a series of targeted events with specific patient groups to provide an open forum for discussion. We will continue to host a series of public awareness events for adults and children.
- Currently children aged over 13 years old can opt out of their health data being used for research, but their voices in PPI/E are seldom heard. We will work with our Young Persons' Advisory Group at Birmingham Children's Hospital, to develop a health data PPI/E theme for young people. The PIONEER team also has strong links with schools and colleges within the region to enable us to access younger stakeholders.
- PIONEER includes a wide range of acute conditions or acute presentation of chronic conditions with many affiliated patient groups and charities. Examples include Sepsis awareness UK, Meningitis now, British Heart Foundation. PIONEER will reach out to these charities to raise awareness of the relevance of PIONEER to their patient group, and seek to work with these organisations to publicise acute health data use and widen PPI/E coverage.
- We will enhance the reach of events by profiling activities on social media, using the UoB/UHB Comms team. We will react pro-actively to national events and news stories, highlighting relevant data opportunities or PIONEER-facilitated research activity, flagging opportunities to inform our strategy.
- We will design and maintain a public Facebook page and Twitter account for PIONEER, to help reach our patients and their friends, to inform them of our research programme and facilitate discussion about health data use
- We will develop PIONEER-affiliated Health Data Ambassadors from within the different ethnic communities represented in the West Midlands to promote research without boundaries. Utilising our extensive links with local and regional communities, ambassadors would be chosen to represent key local communities where research participation is generally low.

PAG will be tasked with developing our long-term PPI/E strategy – and continuing to evolve its principles, delivery and dissemination – utilizing feedback from these groups.

**<u>Key areas for development with our stakeholders to inform the strategy:</u>**

**Theme 1. Increasing awareness and knowledge**

1. To increase patient and public awareness and knowledge of how and why their health data could be used within PIONEER to improve health and care through a series of events and shared information.
2. To ensure we reach as many people as possible, being mindful of the need for diversity and inclusivity
3. To adopt an ageless approach and interact with both adults and children
4. To publish summaries of PPE events and interactions, to openly share what we have learned from our engagement.

**Theme 2. Data into action**

1. To provide patients and the public with real examples of how data use has improved aspects of health and care.
2. To ensure all PIONEER outputs include an accessible summary including summaries suitable for children and adults

**Theme 3. Engagement into involvement**

1. To encourage wider public and patient involvement in PIONEER
2. To reach out to new people for PPI/E interactions, to ensure we are constantly challenged by new voices and opinions

**Theme 4: Evaluating, sharing and adopting PPI/E best practice**

1. To evaluate PPI/E practice using tools such as GRIPP2 reporting checklists
2. To share PPI/E practice with HDR-UK and other Hubs to ensure best practice is adopted
3. To publish PPI/E activity in academic, peer review journals
4. To evaluate the impact and range of PPI/E activities using GRIPP2 checklists



Gallier S, *et al. BMJ Health Care Inform* 2021; 28:e100294. doi: 10.1136/bmjhci-2020-100294
